# Genome Variation in the Model Halophilic Bacterium *Salinibacter ruber*

**DOI:** 10.3389/fmicb.2018.01499

**Published:** 2018-07-19

**Authors:** Pedro González-Torres, Toni Gabaldón

**Affiliations:** ^1^Department of Physiology, Genetics and Microbiology, University of Alicante, Alicante, Spain; ^2^Centre for Genomic Regulation (CRG), The Barcelona Institute for Science and Technology, Barcelona, Spain; ^3^Departament de Ciències Experimentals i de la Salut, Universitat Pompeu Fabra, Barcelona, Spain; ^4^Institució Catalana de Recerca i Estudis Avançats, Barcelona, Spain

**Keywords:** *Salinibacter ruber*, homologous recombination (HR), core genomes, intraspecific diversity, comparative genomics

## Abstract

The halophilic bacterium *Salinibacter ruber* is an abundant and ecologically important member of halophilic communities worldwide. Given its broad distribution and high intraspecific genetic diversity, *S. ruber* is considered one of the main models for ecological and evolutionary studies of bacterial adaptation to hypersaline environments. However, current insights on the genomic diversity of this species is limited to the comparison of the genomes of two co-isolated strains. Here, we present a comparative genomic analysis of eight *S. ruber* strains isolated at two different time points in each of two different Mediterranean solar salterns. Our results show an open pangenome with contrasting evolutionary patterns in the core and accessory genomes. We found that the core genome is shaped by extensive homologous recombination (HR), which results in limited sequence variation within population clusters. In contrast, the accessory genome is modulated by horizontal gene transfer (HGT), with genomic islands and plasmids acting as gateways to the rest of the genome. In addition, both types of genetic exchange are modulated by restriction and modification (RM) or CRISPR-Cas systems. Finally, genes differentially impacted by such processes reveal functional processes potentially relevant for environmental interactions and adaptation to extremophilic conditions. Altogether, our results support scenarios that conciliate “Neutral” and “Constant Diversity” models of bacterial evolution.

## Introduction

The genomic and genetic diversification processes that act on prokaryotic species have been extensively studied (Thomas and Nielsen, 2005; Cohan, 2006; Fraser et al., 2007; Vos and Didelot, 2009; Didelot and Maiden, 2010; Polz, 2013). However, most such studies have historically focused on pathogenic bacteria (Hiller et al., 2010; Morelli et al., 2010; Reeves et al., 2011). In recent years, both whole genome comparisons of isolated strains and metagenomics analyses of environmental samples have opened the way to assess genome variation in free-living species (Mira et al., 2010). The metagenomics approach includes sequences from different individuals of a species within a population, which is used to determine genomic diversity (Coleman et al., 2006; Tully et al., 2015) or the variation of structure and content of genomes across populations, as in the case of *Prochlorococcus* sp. (Luo and Konstantinidis, 2011), marine Crenarchaea (Konstantinidis and DeLong, 2008) and hypersaline environments (Podell et al., 2013). Some of these latter studies have revealed that the genetic diversity of microbial species is often organized in the form of cohesive clusters, each composed of strains with genetic similarity above a given threshold, which are differentiated from other clusters by much larger genetic distances. This cluster structure has been suggested to result from the action of homologous recombination (HR), which would constrain levels of genomic sequence divergence within a population cluster. Such structure is in agreement with the “Neutral Model" (Fraser et al., 2007), which underscores the role of HR as a neutral, passive mechanism that suffices to drive patterns of genetic divergence and diversity. Under this model HR would constrain sequence divergence within a recombining population while increasing the number of transitory unique genetic entities (referred to as the metaclonal scenario).

On the other hand, genome comparisons of multiple strains from the same species, although still limited by the number of available genomes, have allowed exploring the genomic structural diversity and the emerging temporal and biogeographic patterns thereof (Caro-Quintero et al., 2011; Cadillo-Quiroz et al., 2012; Shapiro et al., 2012; Tully et al., 2015). Although scarce, some of these studies have suggested that the environmental genetic pool, involving the flexible genome, is accessible through mechanisms of horizontal gene transfer (HGT) and site-specific recombination. In contrast, the core genome tends to evolve through mechanisms of vertical transmission and HR (Polz, 2013; López-Pérez and Rodriguez-Valera, 2016). In prokaryotes, a considerable fraction of the accessory genome is typically located within genomic islands (GIs), plasmids, and indels (i.e., insertions and deletions). Based on its architecture and evolution, GIs have been recently classified into one of two types: (i) flexible genomic islands (fGI), when they appear in equivalent genomic locations across strains of the same genus or species and (ii) HGT-GIs, when they are strain or clade specific, typically contain viral and transposable elements, and are flanked by tRNAs (López-Pérez and Rodriguez-Valera, 2016). Additionally, fGI were further divided into two categories: additive fGIs, that act as site directed recombination hotspots for the integration of gene cassettes that vary in number and nature, and replacement fGIs, encoded within different gene clusters in each strain, but coding for similar functional structures, generally related to cell envelopes (Rodriguez-Valera et al., 2016).

During the last decade, hypersaline environments have emerged as important models to study prokaryotic ecology, evolution, and adaptive strategies. These environments, defined by salt concentrations above that of sea water, account for 50% of continental waters (Shiklomanov, 1998; Villamor et al., 2017). In addition to these physical–chemical traits, hypersaline environments show particular biotic compositions, including a relatively low number but microdiverse halophilic prokaryotic species, defined as microorganisms whose optimal growth is set over salt concentration of 50 g/l (0.85 M NaCl), or higher and that tolerate 100 g/l salt (1.7 M) (Oren and Mana, 2003; Oren, 2008). In addition, hypersaline environments host the highest concentrations of virus-like particles among aquatic environments (Santos et al., 2012). Therefore they constitute a privileged scenario to study virus–host interactions and adaptations. The “Kill the Winner” model (Thingstad and Lignell, 1997; Thingstad, 2000) is an adaptation of Lotka-Volterra model to understand population fluctuations derived from virus-bacteria interactions in aquatic environments. Complementary to this, the effect of virus-host interaction on the genomic diversity of bacterial populations is the focus of the “Constant Diversity” model (Rodriguez-Valera et al., 2009), which considers the existence of metaclonal populations whose diversity is maintained at equilibrium by means of phage predation.

Among the microbes inhabiting hypersaline environments, the Bacteroidetes *Salinibacter ruber* offers a particularly excellent model for microdiversity studies (Oren, 2013). This species is present in most hypersaline waters worldwide, including salter pond crystallizers, forming part of the core bank, usually as the most abundant bacterial species (Oren, 2013). Furthermore, *S. ruber* has a high intraspecific genomic and functional diversity, at both transcriptomic and metabolomic levels (Rosselló-Mora et al., 2008; González-Torres et al., 2015), even when co-occurring strains are considered (Pasić et al., 2009; Peña et al., 2010; Antón et al., 2013; González-Torres et al., 2015). This makes *S. ruber* an excellent model for microevolutionary studies, particularly with regards to its adaptation to an extreme environment (Oren, 2008, 2013).

Here we set out to broaden our insights into the genomic diversity in *S. ruber*, by comparing eight fully sequenced genomes. Our results define a highly variable accessory genome and an extremely conserved core genome, and highlight the differential impacts that HGT and HR processes have in these two fractions of the genome. Finally, we explore relationships between dynamic regions in the core and accessory genome and adaptation to environmental conditions, including phage predation. Our genomic comparisons, and earlier genomic and experimental studies, underscore the potential value of *S. ruber* as a model for evolutionary genomics studies of bacterial adaptation to hypersaline environments.

## Materials and Methods

### Sample Collection and DNA Extraction

We used in this study eight strains previously isolated from Santa Pola and Campos saltern ponds (**Supplementary Table S1**). Details about the isolation and origin of the *S. ruber* M8, M31, M1, SP38, P13, P18, SP73, and RM158 strains used in this study are provided in **Supplementary Table S1** and in a previous study (Antón et al., 2013). Pre-inocula from individual colonies were set up into 10 ml tubes with 1 ml SW 25% supplemented with 2 g/l yeast extract and grown for at least 10 days at 37°C in an orbital shaker (170 rpm). Finally 500 ml 25% salt medium supplemented with 0.2% yeast extract was inoculated at 5%. Cultures were grown at 37°C in an orbital shaker (170 rpm) to exponential mid-point (DO_600_ = 0,5). Screening and selection of *Salinibacter isolates* were performed as previously described (Antón et al., 2000; Peña et al., 2005). Template DNA was obtained by using the DNeasy Blood and Tissue kit (QIAGEN, Netherlands), according to the manufacturer’s recommendations. The amount and quality of the extracted DNA was checked by spectrophotometry (Nanodrop ND-1000) 1% agarose (Seakem LE FMC Bioproducts) gel and a BioAnalyzer instrument (Agilent) prior to sequencing.

### Sequencing, Assembly and Genome Annotation

Six *S. ruber* genomes (P13, P18, SP38, SP73, RM158, and M1) were assembled *de novo* in this study. Libraries were performed with an Illumina Sample Prep kit according to manufacturer’s recommendations. Sequencing was performed using Illumina Hiseq 2000 (100 pb paired-end read) at the Center for Genomic Regulation (CRG) core facility, Barcelona Biomedical Research Park (PRBB). The generated FASTQ files were first processed to remove poor-quality reads as follows. Reads with a PHRED quality score of <10 were trimmed at the first base and, subsequently, read pairs with any read of length <31 bases were discarded. The remaining reads were assembled using SOAPdenovo 2.04v (Luo et al., 2012), IDBA-UD 1.1.0v (Peng et al., 2012) and JR-Asembler 1.0.4v (Chu et al., 2013) assemblers with varying parameters. For SOAPdenovo, optimal *k*-mer size was set between 41 and 81 bp. For IDBA-UD optimal mink ranged from 40 to 60, min_count from 12 to 20, and min_support from 6 to 20. Gaps were filled with GapCloser from SOAPdenovo2 2.04v (Luo et al., 2012), and the best resulting assemblies (based on N50 and N90 statistics) were combined. Resulting scaffolds were mapped and redirected using progressiveMauve from MAUVE 2.3.1v (Darling et al., 2010) using M31 and M8 *S. ruber* genomes as references (Peña et al., 2010). Formed gaps were closed connecting the scaffolds by using Geneious Read Mapper implemented in Geneious 7.1.7v (Kearse et al., 2012). First, the extremes of resulting scaffolds were used as a reference to drive the assembly toward the gaps. As previously described (Martínez-García et al., 2014), a single file with the paired-end reads was set and used as a query for the mapping driving assembly of the gaps using stringent parameters (no allowed gap between two matched reads, 99% of minimum overlap identity, 1% maximum mismatches per read). A total of 25–100 mapping and extension iterations were carried out until reaching a sequence overlap between the extended extremes of two consecutive scaffolds (i.e., closing the gap). Manual inspection of the overlapping extremes and uniqueness correspondence of the connections were checked by Nucleotide Blast (Altschul et al., 1990). The identity and completion of plasmid sequences was assessed by checking their circularity, the presence of low copy replicative origin (ParA/RepB), and by confirming their lower GC content and higher coverage as compared to the genome average. Additionally plasmidSPAdes pipeline (Antipov et al., 2016) from SPAdes 3.11.1v assembler (Bankevich et al., 2012) confirmed that these contigs conformed plasmidic elements. Strains P13 and P18 were used as negative controls as they lacked plasmids. The raw sequencing data have been submitted to the European Nucleotide Archive (ENA) and Short Read Archive (SRA) and are available under the project accession identifiers PRJEB25455 and SRP151445, respectively. The genome sequences have been deposited at NCBI database under the bioproject accession number PRJNA474853. Biosample accessions are indicated in **Supplementary Table S5**.

*De novo* gene, CRISPR-Cas, and RM systems prediction and annotation were performed using the Integrated Microbial Genomes (IMG) system (Joint Genome Institute) (Markowitz et al., 2012), selecting the algorithm implemented for isolated genomes (IMG/ER). All genomes were annotated with a common platform, including M8 and M31, to reduce possible bias, as it is recommended in comparative genomic studies (Kimes et al., 2014). Functional terms from Clusters of Otologous Groups (COG) (Tatusov et al., 2000) and Gene Ontology (GO) (Ashburner et al., 2000) databases were retrieved using the JGI platform and the Blast2GO software (B2G) 3.1v (Conesa and Götz, 2008; Götz et al., 2008), respectively, (**Supplementary Tables S2, S3**). In the latte, hits with an *E*-value lower than 1 × 10^-20^ and amino acid sequence identity higher than 55% were considered (Ogata et al., 1999). A further characterization of CRISPR-Cas systems and its associated spacers and proteins was performed using CRISPRfinder (Grissa et al., 2007) and Nucleotide Blast (Altschul et al., 1990) against the nr nucleotide collection and specific metavirome and metagenomes from several halophilic environments (**Supplementary Table S4**), including San Diego saltern ponds (EEUU) (Rodriguez-Brito et al., 2010), Tuz Lake (Turkey) (Boujelben et al., 2012), Santa Pola saltern pond CR30 (Spain) (Santos et al., 2010; Ghai et al., 2012; Fernandez et al., 2013), Tyrrell Lake (Australia) (Emerson et al., 2013).

### Genome Comparison Analysis

Genomes of the six strains sequenced in this study and two previously available genomes (Peña et al., 2010) were compared. First, genomes were aligned using the progressive Mauve function from Mauve 2.3.1v (default parameters) (Darling et al., 2010). The output consisted of the alignment showing the Locally Collinear Blocks and the main rearrangements, the sequences contained in the aligned blocks in eXtended Multi-FastA (xmfa format), and the positional orthologs therein (^∗^ort files). The output file was parsed with a Python script which selected the core genome (genes shared between all the strains) and accessory genomes (genes missing in some strains) (Mira et al., 2010). The genome sequences within each species were compared in a pairwise manner using the Nucmer function from the MUMmer package v3.0 (Kurtz et al., 2004). The resulting coordinate files (coords files) were parsed with a python script to calculate the percentage of identity for the aligned sequences. For this, fragments that overlapped over 10% of their length were joined, and those contained within a larger fragment were filtered out. An average nucleotide identity value (ANIb) was obtained for each comparison as the average of each aligned fragment weighted by its fragment length.

### Detection and Functional Characterization of Recombination Events

RDP4 v4.15 (Martin et al., 2010) was used to detect and characterize recombination events. Genomic alignments in ^∗^XMFA format obtained with MAUVE 2.3.1v (Darling et al., 2010) were used as input. Recombination events were predicted in a two step procedure. In a first exploratory phase, four different methods were run: RDP (Martin and Rybicki, 2000), GENECONV (Padidam et al., 1999), MaxCHI (Smith, 1992), Chimera (Posada and Crandall, 2001). In a second phase, the program re-scanned more thoroughly every detected event with seven methods: BootScan (Salminien et al., 1995), SiScan (Gibbs et al., 2000), RDP, GENECONV, MaxCHI, Chimera and 3Seq (Boni et al., 2007). Only recombination signals considered by at least three of the seven methods were considered, using a cutoff value *p* < 0.001 in all cases. For RDP, window size was set to 90 nucleotides and for MaxCHI the variable sites per window was set to 210. For the remaining methods, default parameters were applied. The predicted recombination events were manually curated, and their breakpoints were inferred with MaxCHI method (considered the most accurate breakpoint detection method among the five non-parametric methods in RDP3) (Martin et al., 2010). From these data, the following recombination variables were inferred: recombined fraction of the genome, number of recombination events per strain, and distribution of sizes of recombination events (i.e., length of the recombined region). Nucleotide sequences of the predicted recombined fragments were retrieved from the genome FASTA files using the positional cordinates provided by RDP4 4.15v (Martin et al., 2010). For these sequences, *de novo* gene prediction and annotation was performed as indicated above.

### Evolutionary and Phylogenomics Analysis

We reconstructed the clonal genealogy of the analyzed strains using ClonalFrame v1.2 (Didelot and Falush, 2007), a Bayesian inference method that, uses the genome alignment for the eight strains as input. Only aligned regions containing all genomes and longer than 500 bp were used. Three independent ClonalFrame runs, each consisting of 40,000 iterations were run, and the first 20,000 iterations for each run were discarded as burn-in. The convergence of the tree runs was checked, making sure that they produced consistent estimates of the clonal genealogy, and the global parameters *r*/*m* (were *r* = rate of recombination and *m* = rate of mutation), rho/theta (σ/𝜃) (Vos and Didelot, 2009) (where σ and 𝜃 are the the rates of occurrence of recombination and mutation, respectively) with 95% credibility (Vos and Didelot, 2009). The ratio σ/𝜃 is a measure of the frequency at which recombination occurs relative to mutation, whereas *r*/*m* correspond to the ratio of rates at which nucleotides become substituted as a result of recombination or mutation, thus measuring the relative impact of HR on the observed population genetic diversification.

The amino acid sequences for the positional orthologous genes were retrieved from the GenBank files and pairwise coding sequence alignments between selected strains were performed with MUSCLE 3.8v (Edgar, 2004). The resulting alignments were reverse-translated to codon-based nucleotide alignments using trimAL 1.3v and the corresponding coding sequences (Capella-Gutiérrez et al., 2009). dN/dS values were obtained using the CodeML function, (parwise mode with model 1 NS sites 0 parameters), from PaML package 4.4v (Yang, 2007). A low ratio (dN/dS <1) indicates purifying selection, whereas a high ratio (dN/dS >1) is suggestive of diversifying selection. Finally, pairwise rRNA 16S ANI values were calculated from pairwise aligments using SILVA Incremental Aligner (SINA) 1.2.11v (Pruesse et al., 2012).

### Statistical Analysis

Tests for functional enrichment of genes contained in recombination fragments and GIs vs. the genomic background were performed using the Fisher Exact Test (FT) and the annotated COG and GO terms. Fisher’s test were performed for each of the functional categories in each species, in the case of COG, by applying a FDR (False Discovery Rate) correction and considering a *p*-value < 0.05 as threshold for over or under- representations, compared to the whole genome as implemented in Gossip from Blast2Go (B2G) 3.1v (Conesa and Götz, 2008).

## Results and Discussion

### Genome Sequences

In order to gain further insights into *S. ruber* genome diversity we analyzed the complete genomes of eight different strains, of which six (*S. ruber* M1, SP38. SP73. P13, P18, and RM158) have been sequenced and assembled for this study (**Supplementary Table S5**), and the other two (*S. ruber* M8 and M31), including the reference strain for the species, were obtained from previous studies (Mongodin et al., 2005; Peña et al., 2010). The set includes strains isolated from two mediterranean saltern ponds in Spain: Campos de Mallorca (RM158, M1, M31, and M8) and Bras del Port crystallizer CR30 Santa Pola (P13, P18, SP38 y SP73) (**Supplementary Table S1**). These two locations are situated 320 Km apart from each other and their ecological parameters and microbial communities are well-characterized from previous studies (Ventosa et al., 2014). The strains correspond to serial isolations of the two sites over the course of 10 years. Our assembly strategy produced a single chromosome for each of the strains, and for 18 plasmids present in the various strains (**Supplementary Figure S1** and **Supplementary Table S6**). We next reconstructed whole genome alignments of the eight strains. All strains shared a high level of sequence identity over aligned regions (Tetranucleotides >99.9%, ANIb >98%, and 16S rRNA ANI 98%) (**Supplementary Tables S6, S7**), indicating that they belong to the same genospecies (Konstantinidis and Tiedje, 2005). The absence of a clear biogeographical structure, in terms of the genetic divergence between strains from the two sites, represented also by clonal frame (CF) distributions (detailed below), is in line with earlier results based on Pulse Field Gel Electrophoresis (PFGE) patterns (Peña et al., 2005), and Multi-Locus Sequence Analysis (MLSA) (Rosselló-Mora et al., 2008). To avoid biases, all genomes, including the two from other studies, were annotated (or re-annotated) using a common framework (see section “Materials and Methods”). Thanks to the use of updated databases and methods, the annotation of the previously available strains, M31 and M8, improved with respect to the available version. For instance, for M31 our annotation detects 296 additional genes, and reduces the number of hypothetical proteins from 35 to 24%.

### Pangenome Distribution, Architecture and Overall Conservation of Synteny

**FIGURE 1:**
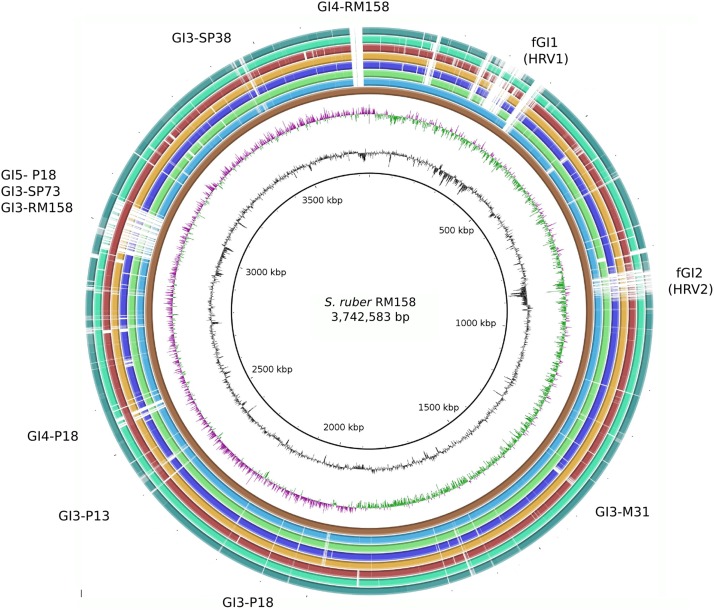
Schematic representation of a genome alignment for the eight *Salinibacter ruber* strains considered in this study. Extensive syntenic core genome regions are represented as collinear blocks interrupted by accessory elements (genomic islands and indels), which are identified in the outer area. Colored height inside each block is proportional to sequence identity level respect to RM158 strain (considered as the reference). From inner to outside ring: genome size (Kbp), GC content, GC skew, *S. ruber* RM158 (dark brown), *S. ruber* M1 (light blue), *S. ruber* M31 (light green), *S. ruber* M8 (dark blue), *S. ruber* P13 (orange), *S. ruber* P18 (maroon), *S. ruber* SP38 (spring green), *S. ruber* SP73 (dark green).

**FIGURE 2:**
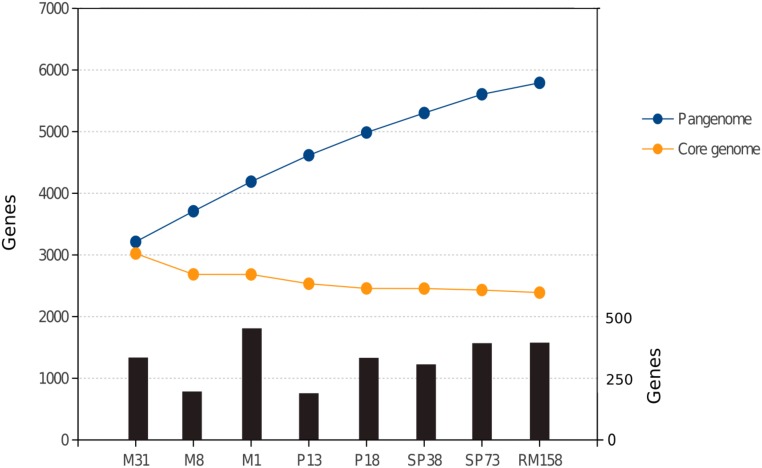
Gene content in the pangenome (blue dots) and stable core genome (orange dots) as *S. ruber* strains analyzed in this study are sequentially considered in a random order. Black bars indicate the number of new genes contributed to the pangenome by each strain (secondary *y*-axis in the right).

The core genome comprised large syntenic blocks that were extremely conserved at the sequence level, and were interrupted by inter-spersed GIs and indels, which together with plasmids comprise the flexible genome (**Figure 1** and **Supplementary Figure S2**). This is in line with observations from other free-living genomic models of aquatic bacteria such as *Prochlorococcus* sp. (Coleman et al., 2006). *Pelagibacter ubique* (Vergin et al., 2007) or *Alteromonas macleodii* (López-Pérez and Rodriguez-Valera, 2016). All vs. all genome comparisons of the eight strains defined a core genome of 2,389 genes, which on average represented 73.53% of the genome. This indicates that *S. ruber* entails higher microdiversity than the bacterial average (Lapierre and Gogarten, 2009), and closer to that of pathogenic bacteria than other free-living extremophiles such as *S. islandicus* (86%) (Cadillo-Quiroz et al., 2012) (**Figure 2**). Our reconstructed *S. ruber* pangenome comprises 5,792 genes (2,389 core and 3,403 accessory genes). Remarkably, most of the expansion of the flexible genome occurs within strains of the same CF, and even between co-isolated strains. This fact is also reflected in the high proportion of strain-specific genes, with each newly sequenced genome contributing about 300 new genes. This shows that *S. ruber* has an open pangenome (**Figure 2**), very similar to what has been reported for *Escherichia coli* (Mira et al., 2010) or *Haemophilus influenzae* (Hogg et al., 2007), which are pathogens with large, diverse, and highly dynamic flexible genomes resulting from their pathogenic lifestyle (Roberts and Kreth, 2014), or some free-living marine species such as *A. macleodii* (López-Pérez and Rodriguez-Valera, 2016). We also found that the plasmid content varied among the strains, both in terms of plasmid type and copy number (inferred from mean relative coverage) (**Supplementary Table S5**).

### Evolution of the Core Genome

**FIGURE 3:**
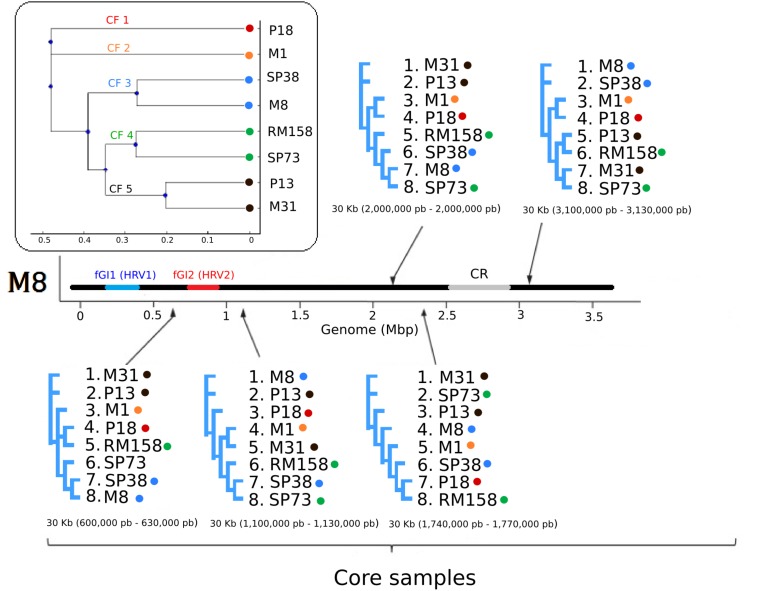
Maximum likelihood trees of the eight *S. ruber* strains included in this study. Members of the five CFs identified (box) were considered and labeled with color-coded dots. The consensus tree based on the whole aligned core genome is shown in the box. The trees surrounding the genome were calculated based on alignments of randomly selected 30 Kb core genome regions, whose relative positions in *S. ruber* M8 are indicated in brackets. In all the cases *S. ruber* M8 was used as a reference genome to locate the position of the sequence used to generate the trees.

We next set out to investigate possible evolutionary mechanisms underlying the above described genomic variability in both the core and the accessory genome. In other aquatic microorganisms such as *Shewanella baltica* or *A. macleodii*, it has been shown that HR shapes the diversity of the core genome (Fernández-Gómez et al., 2012; López-Pérez and Rodriguez-Valera, 2016). In order to explore the impact of HR in the evolution of *S. ruber* core genomes, we estimated the clonal genealogy, as well as r/m and rho/theta (σ/𝜃) ratios (Vos and Didelot, 2009) (see section “Materials and Methods”). Our results suggest that the eight analyzed sequences belong to five distinct CFs. CF is a term that refers to the smallest unit of diferenciation in prokaryotes, (also referred to as strain biotypes or serotypes) and equivalent to the concept of “epidemic clone” used in the clinical field (Cui et al., 2015; López-Pérez and Rodriguez-Valera, 2016; Osaka et al., 2018). Strains within the same CF showed lower ANIb and dN/dS pairwise distances as compared to strains from different CFs (**Figure 3**), which is in line with previous studies in which HR was shown to act as the main evolutionary mechanism in highly recombinogenic bacteria (Castillo-Ramírez et al., 2011; Cadillo-Quiroz et al., 2012). Recombination ratios (*r*/*m*) and (σ/𝜃) values for *S. ruber* were 1.52 and 0.29, respectively, higher than those reported for many other species with different lifestyles (Vos and Didelot, 2009), but similar to other extremophiles, where HR is thought to be one of the main evolutionary forces (Papke et al., 2004; Whitaker, 2005).

Metagenome recruitment analysis (**Supplementary Figure S3**) revealed discontinuities around 95% of ANI, as described by previous studies in marine microorganisms (Luo and Konstantinidis, 2011; Caro-Quintero and Konstantinidis, 2012). The maximum distribution peak is around 98% ANI, well above 95%, considered the threshold for species with cohesive population structures (Cuadros-Orellana et al., 2007; Tully et al., 2015) maintained by HR (Fraser et al., 2007). This 95% identity threshold corresponds with a 70% DNA-DNA hybridization value, above which two strains are considered to be from the same species and under which HR efficiency decreases rapidly (Konstantinidis and Tiedje, 2005; Caro-Quintero and Konstantinidis, 2012; Yarza et al., 2014). Moreover (*r*/*m*) ratio value in *S. ruber*, 1.52, well above the reference value of 0.25 (*r*/*m*), considered the minimum for which HR can act as a cohesive force on the population emerging clusters (Fraser et al., 2007).

Altogether *r*/*m* measures, ANIb and dN/dS among CF support a predominant role of HR in maintaining genome similarity (both at the sequence and the gene order level) in the genomic core regions of *S. ruber.* This high impact of HR would explain previously reported phylogenetic inconsistencies in MLST studies (Rosselló-Mora et al., 2008) and the absence of clear biogeographic patterns (Peña et al., 2005). Similarly, we observed high levels of phylogenetic inconsistency when the rRNA gene tree was compared to phylogenetic reconstructions of randomly selected core genome regions (**Figure 3**). Despite this general trend, some previous studies have detected biogeographical patterns when analyzing metabolomic and HGT events profiles (Rosselló-Mora et al., 2008; Antón et al., 2013; Peña et al., 2014), probably reflecting recently acquired elements from the accessory genome pool and the effect of alternative evolutionary mechanisms acting over this fraction.

### Core Genome Components and Functions Enriched in Recombined Regions

High-salinity environments generally are concomitant with high levels of temperatures, heavy metals, and UV radiation. In these conditions microorganisms have developed strategies that promote the repair of DNA. These include repair and recombination systems (Garcia-Gonzalez et al., 2013; van Wolferen et al., 2013), competence capabilities, as well as defense systems such as restriction modification (RM) (Oliveira et al., 2016) and CRISPR-Cas (Mojica et al., 2005), the last two acting as secondary barriers of DNA integration by means of HR (Thomas and Nielsen, 2005). So far no competence system has been described for *S. ruber*. We detected comEA (*rec2*) in the core genome. This gene is involved in DNA internalization in competent Gram-negative bacteria (Takeno et al., 2011). Internalized DNA can also be exploited as a carbon source, especially in environments such as halophilic media with high concentrations of DNA and viral lysis (Thomas and Nielsen, 2005; Rodriguez-Valera et al., 2009). In addition, we detected the RecFOR repair and recombination system which, similarly to the RecBCD and SOS systems, is able to differentiate between own and foreign DNA by means of *Chi* sequences (Halpern et al., 2007), and which additionally acts as HR-dependent DNA repair system. We detected a low abundance of RM and CRISPR-Cas systems in the core genome. This could be compensated by the presence of an adapted and optimized RecFOR system, as it has been noted in other species (Handa et al., 2009; Vasu and Nagaraja, 2013). RecFOR system could be contributing to the maintenance of species identity, acting as a primary barrier against foreign DNA integration (Jeltsch, 2003).

Genes under positive selection or involved in HGT have been implicated in adaptation to the environment (Yahara et al., 2016). We here explored whether core genome regions involved in HR events are enriched in certain functions (see section “Materials and Methods”). Altogether, we detected 958 HR events (**Supplementary Figure S4**), which collectively covered a 31% of the genome. The number of detected events in pairwise comparisons was congruent with the clonal frame tree topology (**Figure 3**), and the results of ANI, TETRA, and phylogenetic analyses (**Supplementary Tables S6, S7**). Only 233 events (25%) were longer than 10 kb, and 28 (3%) were longer than 50 kb. Four out of the six events longer than 100 kb covered fGI-1, in agreement with previous observations (López-Pérez et al., 2014b; López-Pérez and Rodriguez-Valera, 2016), and with the Constant Diversity model (Rodriguez-Valera et al., 2009) as further disused below. Functional analysis revealed an under-representation of 10 different COG categories (Fisher’s test, *p* < 0.05; FDR <0.05) in genomic regions involved in HR: “Ribosomal structure” (COG J), “Cellular motility” (COG N), “Postranscriptional modification” (COG O) and most of the categories related to cellular metabolism (**Supplementary Table S8**). This underrepresentation of metabolic genes in regions involved in HR is in stark contrast with their preponderance among regions involved in bacterial HGT (Popa et al., 2011). Among “Replication, recombination and repair” (COG L), 58% (29/50) of recombined genes corresponded with “site-specific tyrosine recombinase” (XerD) term, which was the most abundant term among HR regions. These enrichments support a role of transposable elements and *XerD* in HR processes, directly promoting their own mobilization and likely hitchhiking neighboring genes (Thomas and Nielsen, 2005), thereby contributing to genetic exchange between accessory and core genome fractions (Garcia-Gonzalez et al., 2013). Amongst COGs involved in “cellular proceses and signaling” and “cellular metabolism” we found significant enrichments (*P* < 0.05, pFDR < 0.05, Fisher’s test) in functional terms related to glycosylation, “Cell wall/membrane/envelope biogenesis” (COG M) and “Carbohydrate transport and metabolism” (COG G) (**Supplementary Table S8**), which suggest a role of HR in the dynamics of envelope diversity. This includes modifications of surface elements usually encoded within GIs O-antigen clusters of free-living and pathogenic prokaryotes (Dobrindt et al., 2004; Fernández-Gómez et al., 2012; Rodriguez-Valera et al., 2016) and over syntenic clusters in the case of *S. ruber* fGIs as described below. Complex envelopes with a variable repertoire of glycosylases, together with the presence of the RecFOR system, detailed above, may compensate for the low abundance of RM and CRISPR-Cas systems in *S. ruber*, and could act as major defense systems against viral infection (Martin-Cuadrado et al., 2015; Rodriguez-Valera et al., 2016). O-antigen diversification has been described as an adaptive mechanism in response to environmental viral pressure in free living bacteria (López-Pérez et al., 2013) or to the host immune system in pathogenic bacteria (Schmidt et al., 2003; Wang and Quinn, 2010). Thus, recombination could sustain the high levels of inter-strain diversity observed in previous *S. ruber* metabolomic and transcriptomic studies (Rosselló-Mora et al., 2008; Antón et al., 2013; González-Torres et al., 2015). Additionally, we found enrichments in functional terms related to resistance, pathogenesis and adaptive mechanisms linked to “coenzyme and inorganic ion transport” (COG H and COG P), and “defense mechanisms” (COG V). Among the enriched GO terms, we detected multidrug or antibiotic resistance, transport systems, or beta-lactam resistance (**Supplementary Table S9**), which modulates intraspecific cellular communication or competition in several bacteria, including *S. ruber* (Bernier and Surette, 2013; González-Torres et al., 2015). Over 70% of recombined genes within COG V category were related to multidrug resistance or resistance to antibiotics, which in *S. ruber* are associated with intraspecific cellular communication processes or competition (González-Torres et al., 2015). Among transport systems, we found enrichments in ABC transporters, and Fe and TonB external receptors, which would suggest positive selection on HR events that affect transport capacities, or environmental adaptation under iron-limiting conditions as described in both free-living and pathogenic human bacteria (Hacker and Carniel, 2001; Gonzaga et al., 2012; Yahara et al., 2016).

### Flexible Genome: Diversity and Architecture of GIs

We observed a large microdiversity of the accessory genome, exemplified by the variable number (2–5), size (10–200 kb), gene content, and structure of GIs among *S. ruber* genomes (**Table 1**). The genomic fraction represented by GIs ranged from 6.1 to 10.6%, which is larger than those observed in many other *Bacteroidetes* and bacterial classes (2–5%) (Fernández-Gómez et al., 2012). As usual (López-Pérez and Rodriguez-Valera, 2016), these regions presented lower GC content and Codon Adaptation Index (CAI) values than the genome average, and were under-represented in environmental recruitments (**Supplementary Figure S5**), reported also in the case of *S. ruber* M8 and M31 strains (Pasić et al., 2009; Peña et al., 2010). Here, the availability of a larger number of genomes from different CFs allowed us to characterize these GIs under a functional and structural perspective. We confirm the presence of two types of GIs, as reported for other bacteria (Fernández-Gómez et al., 2012; López-Pérez et al., 2014a). Firstly, fGIs appeared as two regions (fGI-1 and fGI-2) located in the same relative position, corresponding to hypervariable regions (HRV-1 and HRV-2) (Peña et al., 2010). Secondly, a variable number of HGT-GIs, indels, and plasmids, which are mostly strain-specific but sometimes shared among few strains (**Supplementary Figure S5**).

**Table 1:** Characteristics of the genomic islands found in the eight *Salinibacter ruber* strains compared in this study.

Genome	GI Classification	GI ID	Number ORFs	Size (kb)	Initial position (bp)	Final position (bp)	%GC	%Genome covered
M8 M(1999)	fGI	fGI1 (HRV1)	127	151.71	249,507	401,287	61.3	3.96
	fGI	fGI2 (HRV2)	134	129.92	829,018	958,941	58.2	3.39
	fGI	fGI1 (HRV1)	111	134.12	223,961	358,080	62.1	3.74
M31	fGI	fGI2 (HRV2)	82	88.14	774,703	862,838	59.1	2.46
M(1999)	HGT-GI	GI3-M31	44	42.75	1,360,489	1,404,241	55.8	1.19
M1 M(1999)	fGI	fGI1 (HRV1)	151	177.85	235,218	413,070	60.5	4.72
	fGI	fGI2 (HRV2)	44	51.25	841,588	892,833	62.2	1.36
P13 SP(1999)	fGI	fGI1 (HRV1)	139	140.56	246,865	387,420	61.9	3.90
	fGI	fGI1 (HRV1)	77	74.90	819,096	893,996	60.8	2.08
	HGT-GI	GI3-P13	30	27.30	2,335,400	2,362,700	50.6	0.76
P18	fGI	fGI1 (HRV1)	87	109.18	237,245	346,422	62.7	2.92
SP(1999)	fGI	fGI2 (HRV2)	50	56.79	793,772	850,557	62.4	1.59
	HGT-GI	GI3-P18	18	33.80	1,990,500	2,024,300	58.7	0.90
	HGT-GI	GI4-P18	35	43.50	2,544,500	2,588,000	66.0	1.16
	HGT-GI	GI5-P18	105	114.61	2,608,780	2,723,390	61.4	3.06
SP38	fGI	fGI1 (HRV1)	145	168.38	230,386	398,753	62.5	4.55
SP(2006)	fGI	fGI2 (HRV2)	42	50.10	817,511	867,607	62.5	1.35
	HGT-GI	GI3-SP38	26	23.80	3,584,200	3,560,400	51.0	0.64
SP73 SP(2006)	fGI	fGI1 (HRV1)	205	196.16	256,113	452,273	60.4	5.03
	fGI	fGI2 (HRV2)	103	104.73	872,601	977,331	58.6	2.69
	HGT-GI	GI3-SP73	106	113.67	2,993,000	3,106,670	61.4	2.91
RM158	fGI	fGI1 (HRV1)	127	156.47	249,611	406,078	60.7	4.03
M(2006)	fGI	fGI2 (HRV2)	97	94.72	840,001	934,723	58.7	2.44
	HGT-GI	GI3-RM158	102	113.68	2,943,003	3,056,685	61.5	2.93
	HGT-GI	GI4-RM158	18	27.64	3,700,270	3,727,910	57.6	0.72

**FIGURE 4:**
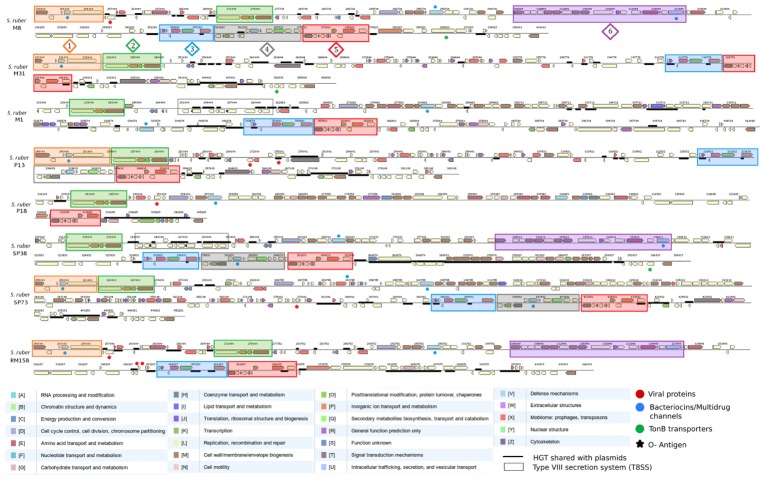
Comparison of the genomic architecture, gene content and cluster distribution, of fGI1 (HRV1) among the eight considered strains. Genes are colored based on their COG-based functional classification and relevant events are labeled with different symbols: viral proteins (red dots), multidrug/bacteriocins (blue dots), heavy metal resistance and tonB transporters (green dots) andO-Antigens (o-chain) (black stars). Six different syntenic clusters or cassettes were identified and delimited in numbered color filled boxes. Regions shared with plasmids appears as underlined. Structural similarities between M8 and RM158 fGI-1 suggest extensive HR from a related strain are usually followed by insertions from plasmids as in the case of the 7 Kb sequence in 3′ RM158 fGI-1 extreme homologous to RM158-pSR67. A similar case is represented for M31 and P13 fGIs, that presented the main differences in 5′ fGI-1 extreme.

The flexible genomic Islands IGF-1 and IGF-2 contained mainly genes from the accessory genome (**Figure 1** and **Table 1**) and only 13 and 15 core genome genes over syntenic clusters or cassettes (**Figure 4**), respectively, as it is often the case in additive fGIs (López-Pérez et al., 2013). These syntenic cassettes, sometimes shared by all strains, were interspersed by transposable elements, genes of viral origin, TonB transporter genes, and TonB-dependent receptors. Some of these TonB receptors are TonB-linked outer membrane proteins that belong to protein families restricted to the Bacteroidetes lineage such as the SusC/RagA (IPR023996) family. Transporter complexes including these outer membrane proteins are involved in the transport of outer membrane proteins and carbohydrates (glycans) in association with SusD proteins, that bind glycans to the cell surface and facilitate shuttling to the TonB-dependent porin (Bolam and Koropatkin, 2012). Additionally, we found functionally conserved blocks with different gene content, usually located in the same areas within the replacement fGIs-2 (**Supplementary Figure S6**). These latter clusters shared only one common protein, as exemplified by a bacteriocin cluster or an O-antigen cluster (**Supplementary Figure S6**). Amongst fGIs we detected an enrichment (*p* < 0,05, FDR < 0.05, Fisher’s test) in genes annotated as “cell wall and membrane envelope biogenesis” (COG M) and “Mobilome: prophages, transposons” (COG X), corresponding mainly to transposable elements and hypothetical proteins (58%), (**Supplementary Table S10**). The high content in hypothetical proteins and transposable elements is associated with the presence of new genes acquired by HGT during microbial adaptation (Dobrindt et al., 2004; Hsiao et al., 2005; Roberts and Kreth, 2014) and recent duplications in *S. ruber* (González-Torres et al., 2015). Enriched COG M genes, mainly coding for sulfotransferases and glycosyltransferases, and located within the O-antigen cluster, are involved in processes of recognition and immunospecificity virus–host recognition (Peña et al., 2010; Wang and Quinn, 2010; López-Pérez and Rodriguez-Valera, 2016). The high diversity and intraspecific genome dynamics of surface genes was consistent with recent metabolomic studies in *S. ruber* (Rosselló-Mora et al., 2008; Antón et al., 2013) and the known high adaptive pressure exerted by viruses from the environment (Santos et al., 2010; Martin-Cuadrado et al., 2015; López-Pérez and Rodriguez-Valera, 2016). Differences in cell surfaces affect susceptibility to phages and phage-host range (Peña et al., 2010; Villamor et al., 2017) an thus result in selection either by differential lysis impact or by processes of signal transduction, according to the “Constant Diversity” model, and in favor of a metaclonal scenario (Rodriguez-Valera et al., 2009). Bacteriocin and multidrug resistance proteins (including betalactamases) have been associated to molecules mediating signaling between bacterial populations in nature (Davies, 2006; Bernier and Surette, 2013). Indeed, efflux pumps, drug resistance, and antibiotic related pathways have been associated with adaptive mechanisms of expression response that mediate intra-specific interactions between co-occurring strains of *S. ruber* (González-Torres et al., 2015). Thus the mechanisms that modulate the content of such elements in the accessory genome could play important roles in adaptation to halophylic environments.

Most HGT-GIs were short (22–45 kb) and strain specific (**Supplementary Figure S7** and **Supplementary Table S1**), with the exception of three (GI5-P18, GI3-SP73, and GI3-RM158), which were homologous and syntenic, and spanned a large region of 113 Kb. All HGT-GIs were flanked by tRNAs, repetitive regions, XerD or phage-related recombinase, which potentially facilitates integration and excision in HGT events (Hacker and Kaper, 2000). The five smaller HGT-GIs were enriched for hypothetical proteins. GI-4P18 (43 kb) contained many membrane genes (COG M), some involved in resistance to organic solvents, as well as transcription factors (COG K), including sigma 70. The three larger HGT-GIs (GI5-P18, GI3-SP73, and GI3-RM158) presented an identical content, except for a 3 kb-long indel generated by transposition in the GI3-SP73. This GI, integrated in a previous event next to an indel present in all the strains (in the case of P18 in a reverse direction), contained several genes coding for ion or carbohydrate transporters (COG P and COG G, respectively) and structural membrane elements (COG M). Many of these transporters code for proteins involved in heavy metal resistance (Cu, Mg, Au, Co, Zn, Cd) and cofactor transport (Zn, Fe, Co). Hence, this GI could act as a metallorresistence island, by analogy to the halophilic island described in M31 (Mongodin et al., 2005). Incidentaly, it could represent a potential bioremediation element. Heavy metal resistance elements are commonly associated to GIs, mobile elements and adaptive processes (López-Pérez et al., 2013; Bellanger et al., 2014; Martin-Cuadrado et al., 2015).

### Plasmid Diversity and Plasmid-Chromosome Genetic Exchanges

A total of eighteen plasmids were present in the analyzed strains, in variable numbers (0–4 per strain) and sizes (10–116 Kb). Plasmids showed a lower GC content as compared to the genome average, and displayed the characteristic low copy replicative origin (ParA/RepB) (Pinto et al., 2012; Ietswaart et al., 2014) (**Supplementary Figure S1** and **Supplementary Table S5**). Similarly to GIs, plasmids showed a high proportion of hypothetical proteins (35%) as compared to the whole genome (chromosome and plasmids) (24%). Smaller plasmids contained the greatest proportion of short ORFs, and of viral recombinase, XerD or terminase (as in case of RM158-pSR31 with 45 ORFs in 31 Kb) (**Supplementary Figure S9**). Larger plasmids contained barrier elements involved in DNA exchange such as CRISPR-Cas, RM systems, secretion systems, as well as membrane elements (**Supplementary Figures S8, S9**). M8-pSR61 plasmid, pSR66-M1, M8-pSR84, and RM158-pSR67 contained clusters of genes coding for type VIII secretion system (T8SS, like curlin) present also in M1 and SP38 fGI-1 (**Supplementary Figures S8, S9**). The presence of secretion systems in virulence or conjugative GIs and plasmids is common among different ecological strategies (Fernández-Gómez et al., 2012; Melville and Craig, 2013). Finally the M8-pSR84 plasmids, RM158-pSR60 and M1-pSR116 contained many genes associated with membrane elements (COG M), some linked to the O-antigen cluster described in the fGIs-II, as well as ionic transporters (COG P) related to resistance to heavy metals and iron transport. We also detected the presence of genes clearly connected with halophylic adaptation such as bacteriorhodopsin (Oren, 2013) in plasmid M1-pSR116. The presence of these common elements (O-antigen and T8SS) between fGIs and plasmids suggests that the former could serve as the transmitter vehicle, eventually incorporating sequences to the GI. The comparison of this same plasmid, M8-pSR84, with fGIs-1, allowed us to identify in fGIs-1of SP38 a 30-kb region with high identity (93%, *e*-value = 0) and synteny. In particular the two last mentioned plasmids, M8-pSR84 and RM158-pSR67, shared a 10 Kb-long syntenic region with a 99.6% sequence identity (block 3, **Supplementary Figures S8, S9**) that included T8SS. The above results suggest that plasmids act as a dynamic vehicle for access to the environmental pool, while the GI would be the gateway to the chromosome.

**FIGURE 5:**
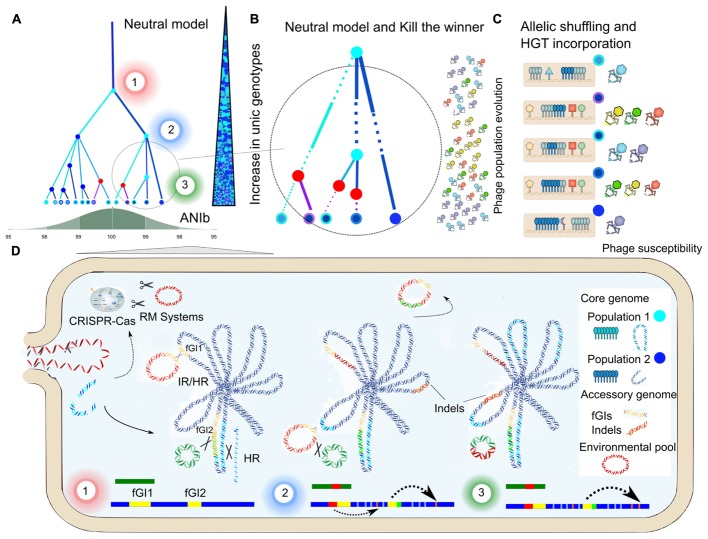
Genome dynamics and ecological models. Schematic representation of: **(A)** the cohesive effect of HR, as predicted by the Neutral Model, limits sequence variation in two *S. ruber* populations (dark blue and clear blue dots). The number of unique genotypes (represented as dots outlined and filled with different colors) increases over time due to the core genome shuffling, although ANIb is maintained over 98% due to the cohesive effect. Significant HR events are highlighted as colored nodes, in which the color indicates the donor (red nodes refers to HGT gene incorporation). Numbered from 1 to 3, different snapshots over the population tree represented in **(D)**. **(B)** Detail of the population tree represented in **(A)**, incorporating the simultaneous effect of phage predation. Branch thickness is proportional to clonal frame abundance, which is modulated periodically by phage populations, as predicted by the Kill the Winner model. **(C)** Allelic shuffling in core genome genes (blue) and phage susceptibility, resulting from glycosyltransferases and surface receptors content (colored with the same code of the corresponding phage). **(D)** Detail of the population tree represented in **(A)**, representing the genome evolution (main chromosome in dark blue and plasmid in green) of a clonal frame from the population 2. Exchanges between clonal frames, by means of illegitimate recombination (IR) or homologous recombination (HR), would be modulated according to the contents in RM and CRISPR-Cas systems. Genomics Islands (GIs) act as gateways of elements of the accessible pool (colored in red) through the integration of plasmidic elements and HR in case of syntenic clusters, contributing to glycosyltransferases. The bars at the bottom represent the linearised genome regions (chromosome in dark blue and plasmid in green) and the recombined regions in a similar way that in the **Supplementary Figure S4**.

To further investigate this possible mechanism, we compared the sequences of each plasmid against the genomes of the other strains, and identified plasmidic regions integrated in the chromosomes. Approximately 50% of hits occurred at fGIs-1 and IGF-2, which represent a small fraction of the genome (6.1–10.6%) (**Supplementary Figure S10**). This would explain that strains of different CFs and geographical origins can share accessory genome sequences. Similar findings have been reported for different species (López-Pérez et al., 2013, 2017). Although we failed to find Integrative and Conjugative Elements (ICEs) in the plasmids, we did find some elements coding for proteins involved in integration and excision, including site-specific tyrosine recombinases (XerD) and integrases. The presence of the genes *comEC* and *comF* in all *S. ruber* strains, suggests that transformation and transduction may provide alternative mechanisms for the transfer of plasmids, as suggested for *A. macleodii* (López-Pérez et al., 2017). Incorporation of new genes in GIs can play a role in modulation of transcription and signal transduction (Coleman et al., 2006). In this respect, the translocation of a gene from a plasmid to a GI could result in increased expression, as some regions in GIs such the O-antigen gene have a much higher expression than plasmids (González-Torres et al., 2015). The rest of plasmids showed peaks of identity between 86 and 94%, which could indicate that these sequences are so ubiquitous in various species that they may have exchanged sequences from other replicons, as observed previously (López-Pérez et al., 2013). In addition, HR events extensively involved fGIs (fGIs-1 and fGIs-2 to 4), as detailed in **Figure 4**, and included events longer than 100 Kb that involved different CFs, in agreement with the Constant Diversity model and metaclonal scenario (Rodriguez-Valera et al., 2009). In some cases HR events affected short syntenic clusters (5–12 kb), and resulted in phylogenetic incongruencies (**Supplementary Figure S11**) (Fernández-Gómez et al., 2012). Altogether, these results underscore the complex intraspecific dynamic network regulating the access to the accessory gene pool in *S. ruber.* The emerging picture is that of plasmids exchanging genetic material with GIs, and these acting as gateways or hotspots, driving exchanges with the rest of the genome including syntenic regions. Hence, plasmids would serve as an important vehicle contributing to genome dynamics, in which the barrier elements encoded by them would modulate inter and intraspecific genetic flows in a strain, thereby affecting its capabilities and abundance (**Figure 5D**).

### The Influence of RM and CRISPR-Cas Systems Evolution on DNA Exchanges

The presence of RM and CRISPR-Cas systems in the accessory genome reflects the complex interactions that exist between gene exchange processes (Corvaglia et al., 2010; Fernández-Gómez et al., 2012; Bellanger et al., 2014). A clear example is provided by the presence of the IC-prr type MR system in the chromosomes of M1, P13, and SP38 strains, in equivalent positions, flanked by XerD (**Supplementary Figure S12**). This unique indel flanked by a recombinase located in an otherwise conserved region, suggests a provenance from accessory regions (plasmids or GIs) (**Figure 5**). In the case of M1-pSR61 plasmid, we also identified an additional RM system, type 1A/B. The presence of different sets of RM systems among strains in a population determines access to the environmental pool and intraspecific exchanges favoring incipient speciation (Cadillo-Quiroz et al., 2012). In this sense, *S. ruber* strains in the same CF presented the same RM system content, and similar HR levels, which were higher in those strains that contained a RM system. This finding supports the idea that there are limits to intraspecific exchanges between strains coding for different RM type systems (Corvaglia et al., 2010). Additionally, we detected the presence of complete CRISPR-Cas systems in the plasmids M1-pSR66 (subtype I-b of halophilic) and SP73-pSR118 (subtype IE *E. coli*) (Makarova et al., 2011) with 38 and 105 spacers, respectively (**Supplementary Figure S13** and **Supplementary Table S11**), as well as additional minor CRISPR arrays in plasmids from these same strains that could act in trans. The CRISPR-Cas system detected in SP73 was syntenic to the *Rhodothermus marinus* R-10 (DSM4254) (Nolan et al., 2009), a species phylogenetically related to *S. ruber.* The presence of these elements in the accessory genome could indicate an adaptive role against phage predation in extreme environments (Mojica et al., 2000; Sorek et al., 2008). Comparisons of spacers against halophylic metagenomes/metaviromes and *S. ruber* (Villamor et al., 2017) and *Haloquadratum walsbyi* (Martin-Cuadrado et al., 2015) halophage sequences confirmed this fact (**Supplementary Tables S4, S11**), associating a viral or prokaryotic protospacer in almost 70% of the cases in M1 (**Supplementary Figure S13**). Two M1 spacers and another from SP73 hit contig 170 from Santa Pola crystalizer CR30 metavirome (Santos et al., 2010). The independent acquisition of spacers for the same mobile element, and the lack of shared spacer sequences between both strains, suggest that, although Mallorca and Santa Pola saltern ponds share a pool of viruses, both viral populations presented biogeographical differences promoting specific adaptations. The presence of M1 spacers whose precursor exogen sequence, protospacer, corresponds to isolated viruses has been related to lower infection susceptibilities (Villamor et al., 2017). Acquisition of independent resistance among CFs would prevent the clonal sweep in periodic episodes, limiting the loss of diversity (Lythgoe and Chao, 2003; Held et al., 2010). Moreover, we found a remarkable number of hits (75) in Lake Tyrrell metavirome of 2010, surpassing the number detected in 2007 (5) in these same ponds (**Supplementary Figure S13**). These findings support the persistence of spacers from viruses that may have had periodic abundance fluctuations within a single pond, fitting the virus-host dynamics predicted by the “Constant Diversity” model under a metaclonal scenario (Rodriguez-Valera et al., 2009).

## Conclusion

Our comparative analysis of eight whole *S. ruber* genomes revealed a diverse and open pangenome, in which each strain presented a unique plasmid and GI profile. In contrast, the core genome is extremely conserved in terms of sequence identity and gene order. We show that HR constitutes the main evolutionary force acting on core genomes. Indeed HR affects over 30% of the genome, breaks clonal structures, and limits sequence divergence within population clusters. Moreover the presence of transposable elements, XerD, recombinases and phage terminases, also abundant in fGIs, HGT-GIs and plasmids suggested a complete and dynamic network of genetic exchange between the accessory and the core genome by means of homologous or illegitimate recombination (**Figure 5**). In this model, GIs act as gateway of elements of the accessible pool through the integration of plasmidic elements and HR in case of syntenic clusters, contributing to glycosyltransferases and metabolic capacities diversity through transfection or transformation. Exchanges between clonal frames would be modulated according to the contents in RM and CRISPR-Cas systems.

The observed HR evolutionary parameters, together with genomic traits (ANIb and dN/dS) and data from metagenomics recruitments, fitted with Fraser’s Neutral model (Fraser et al., 2007), reflecting the cohesive effect of HR over *S. ruber* populations maintaining the species identity. This model highlights the role of HR as a passive, neutral mechanism driven solely by genetic divergence. However, we detected functions that are over-represented in genes that formed part of recombined regions. In many cases these over-represented functions are potentially related to adaptations to the halophilic niche inhabited by *S. ruber*, suggesting the influence of natural selection. These include, among others, accessory genes involved in intraspecific cellular communication, multidrug resistance, ionic transport, as well as genes involved in glycosylation and thereby in resistance to phage predation. Resistance to phage predation is the key trait considered by the “Kill the Winner” model, which contemplates frequency-dependent selection on bacterial phage-susceptibility types as mediated by phages. It has been suggested that periodic phage predation dynamics may be considered simultaneously with other evolutionary models (Rodriguez-Valera et al., 2009) and also that different ecological and evolutionarily processes may play an important role on parallel trajectories under a metaclonal scenario (Good et al., 2017).

We consider that our results provide support for a synergistic action of processes postulated by both the Neutral and the Constant Diversity models. Our proposed framework (**Figure 5**) considers a metaclonal scenario, in which core and accessory genomes would be differentially affected by HR and HGT, resulting in the observed high microdiversity levels in *S. ruber*, while preventing clonal sweep and limiting sequence variation within clonal frames. Both, biotic (viral) and abiotic conditions (temperature, UV radiation, oligotrophy and salinity) justify the presence of recombination machinery and the high levels of HR, which shuffles the content of both the core and flexible genomes. The coexistence of several clones with different characteristics in a population would dilute the selective pressure by phages and would result in a more efficient exploitation of the scarce resources in a stressful environment, as suggested by the Black Queen hypothesis (Morris et al., 2012). This equilibrium could be maintained by a phage population that would prevent any clonal sweep or periodic selection event. Altogether, the high diversity and genome dynamics uncovered by the comparison of the first eight genomes from *S. ruber* highlights the suitability of this species as a model for the study of genome micro-evolution and speciation in hypersaline environments.

## Author Contributions

TG supervised the project. PG-T and TG planned the sequencing and bioinformatics analysis and wrote the manuscript. PG-T performed the bioinformatics analyses.

## Conflict of Interest Statement

The authors declare that the research was conducted in the absence of any commercial or financial relationships that could be construed as a potential conflict of interest.

## Supplementary Material

FIGURE S1 Representation of 24 replicons (8 chromosomes and 18 plasmids) present in the eight strains in this study. The main traits (nomenclature, genome size, gene and GC content) are detailed for each genome. From inner to outside ring: Ring 1: represents GC skew index (GCSI) defined as (G-C/C+C) (green and purple); Ring 2: GC content (%) (colored in black). Genes appears represented in external rings and colored based on clusters of orthologous groups (COGs) functional classification. Colors corresponds to those associated to each strain in **Figure 1**.

FIGURE S2 Chromosome alignment representation for the eight *S. ruber* strains considered in this study. Extensive syntenic core genome regions appears represented as collinear blocks interrupted by accessory elements; genomic islands and indels. Colored fraction inside each block is proportional to the sequence identity level with respect to M8 strain.

FIGURE S3 Metagenomic recruitment and discrete populations. Coverage plot representing fragment recruitment of the **(A)** Santa Pola CR30 crystallizer (Spain) (Fernandez et al., 2013); **(B)** Lake Tyrrell metagenome (Australia) and **(C)** two different San Diego High Salt ponds metagenomes (United States) against *S. ruber* main chromosomes and **(D)** Santa Pola CR30 crystallizer (Spain) (Ghai et al., 2011) against plasmids sequences. For each graph, the coverage (*y*-axis) is normalized to the length of the reference and represents the relative *in situ* abundance of the corresponding population.

FIGURE S4 Homologous recombination map. Genomic map representing the inferred recombination events among the considered strains. Each strain appears associated to a color code (top bar) consistent with **Figure 1** and **Supplementary Figure S1**. The bottom bars represent the recombination events, which are colored according to the predicted donor strain. FGI1, fGI2 and the conservated region (CR) described in previous studies (Peña et al., 2010) are highlighted in the figure.

FIGURE S5 Metagenomic recruitment and GI identification. Alignment of the eight *S. ruber* chromosome sequences with metagenomic sequences from Santa Pola saltern pond, CR30 crystallizer (Spain) (Ghai et al., 2011). Values on the *y*-axis indicate nucleotide percent identity, usually over 95%. Areas with unusually low representation in the metagenome corresponded to under-recruiting genomic islands: fGIs1 and fGIs2 (indicated in blue and green, respectively) and HGT-GIs in green.

FIGURE S6 Overall comparison of the genomic architecture, gene content and clusters distribution, of fGI2 (HRV2) among the eight analyzed strains. Regions shared with plasmids are underlined. Six different syntenic clusters or cassettes were identified and delimited in numbered colored boxes. Gene colors and symbols were consistent with **Figure 4**.

FIGURE S7 Gene content and functional traits of HGT-GI islands among the eight analyzed strains. RM systems are delimited with red boxes and tRNA genes are labeled with triangles. Gene colors and symbols were consistent with **Figure 4**.

FIGURE S8 Gene content and functional traits of plasmids detected amongst three of the eight strains analyzed in this study (*S. ruber* M8, *S. ruber* M31, *S. ruber* M1). RM systems, type 8 secretion systems (T8SS) and CRISPR-Cas systems are delimited in boxes and genes are labeled with a triangle and appear flanking HGT-GI islands. Three different events were identified and delimited in numbered color filled boxes representing three cases of extensive homologous regions involving plasmids of four different strains: M8 (pSR84), SP73 (pSR118), SP38 (pSR76), and RM158 (pSR60, pSR67). Gene colors and symbols were consistent with **Figure 4**.

FIGURE S9 Gene content and functional traits of plasmids detected amongst three of the eight strains analyzed in this study (*S. ruber* SP73, *S. ruber* M38, and *S. ruber* RM158). RM systems, type 8 secretion systems (T8SS) and CRISPR-Cas systems are delimited in boxes and genes are labeled with a triangle and appear flanking HGT-GI islands. Three different events were identified and delimited in numbered color filled boxes representing three cases of extensive homologous regions involving plasmids of four different strains: M8 (pSR84), SP73 (pSR118), SP38 (pSR76), and RM158 (pSR60, pSR67). Gene colors and symbols were consistent with **Figure 4**.

FIGURE S10 The main bar represents the cumulative percentage (*y*-axis) of 18 plasmid sequences, included in the genome of six *S. ruber* strains (*x*-axis), that presented homology with chromosome sequences among the eight different *S. ruber* strains (colored in the legend). Among these sequences, an additional unique secondary bar per strain represents the percentage of total sequences that presented homology with fGI1 (in red) and fGI2 (in black). For P13 and P18 strains (values not represented), the last distributions were 37.11 and 4.6% for fGI1 and 42.26 and 14.81% for fGI2, respectively.

FIGURE S11 ML trees of 6 of the 10 syntenic clusters or cassettes shared by at least four strains and described in fGI1 and fGI2 (**Figure 4** and **Supplementary Figure S3**). Strains are indicated in all the branches and clonal frames (CFs) correspondence indicated by colored dots consistent with **Figure 4**. The line segment at the bottom part of each tree shows the branch length scale (substitutions/site units).

FIGURE S12 RM systems diversity and microevolutive mechanisms. **(A)** Genome alignment showing the syntenic genomic environment (underlined in red) interrupted in case of *S. ruber* M1, SP38 and P13 strains by an indel (underlined in back) that contained a type 1C-prr RM system, flanked by tirosin recombinases XerD, likely involved in the chromosome integration. **(B)** Most common RM systems present in bacteria (upper part) and those present amongst *S. ruber* strains (bottom).

FIGURE S13 CRIPR-Cas systems microdiversity. **(A)** CRISPR- Cas systems found in *S. ruber* SP73 and *S. ruber* M1 plasmids, indicating the type and cas genes content and spacer sequence. CRISPR arrays were represented with dotted red lines. **(B)** Comparison and syntenic similarities between CRISPR-Cas system found in *S. ruber* SP73 strain and its best hit in *Rhodothermus marinus* DSM 4252 strain. **(C)** Distribution of the main protospacer hits amongst environmental metagenomes (yellow), metaviromes (red), and unknown (blue). **(D)** Number of hits found mean protospacer recruitment versus lake Tyrrell Site A and B metaviromes from 2007 and 2010 years.

TABLE S1 General features of the analyzed genomes: Conal frame (CF); genome size [Size (Mbp)], number of genes (Total ORFs); strain specific genes (Unique genes); number of plasmids (Plasmids), number of flexible genomic islands (fGIs), number of unique islands (HGT-GIs), isolation data (MLL; Campos saltern ponds: SP: Santa Pola saltern pond, CR30 crystallizer), assembly status, G + C content and tRNA gene content (tRNAs genes).

TABLE S2 Gene distribution based on Cluster of Orthologous Genes (COG) classification over whole genomes.

TABLE S3 Functional genomic and genetic statistics for the eight *S. ruber* strains annotated in JGI IMGER (Integrated Microbial Genomes- Joint Genome Institute) platform.

TABLE S4 Metagenome and metavirome datasets considered in this study. Some characteristics, reference and analysis performed in this study were indicated.

TABLE S5 Sequencing parameters and coverage among replicons assembled in this study.

TABLE S6 16S rRNA ANI (%) values for pairwise comparisons among strains in the study are given in the upper triangle and the ANIb (%) for genome comparisons are given in the lower triangle.

TABLE S7 Tetranucleotide (%) pairwise values (TETRA) among the strains in this study are given in the upper triangle and the dN/dS (%) value between genomes are given in the lower triangle.

TABLE S8 Fisher’s Exact Test detecting over or under representation of COG functions in recombined events respect to the rest of the genome.

TABLE S9 Fisher’s Exact Test detecting over or under representation of GO specific terms over general COG functions in recombined events respect to the rest of the genome.

TABLE S10 Fisher’s Exact Test detecting over or under representation of COG functions contained in fGIs respect to the rest of the genome over 8 *S. ruber* strains.

TABLE S11 Spacer sequences, ID and associated hits (number) to public NCBI databases (red) diverse environmental halofilic metaviromes (blue), metagenomes (green). In some cases, candidate protospacers were indicated.
