# Supplementary figures and images for: Genome Variation in the Model Halophilic Bacterium Salinibacter ruber
Source: Front Microbiol. 2018 Jul 19;9:1499. doi: 10.3389/fmicb.2018.01499 (PMC6060240; doi:10.3389/fmicb.2018.01499)

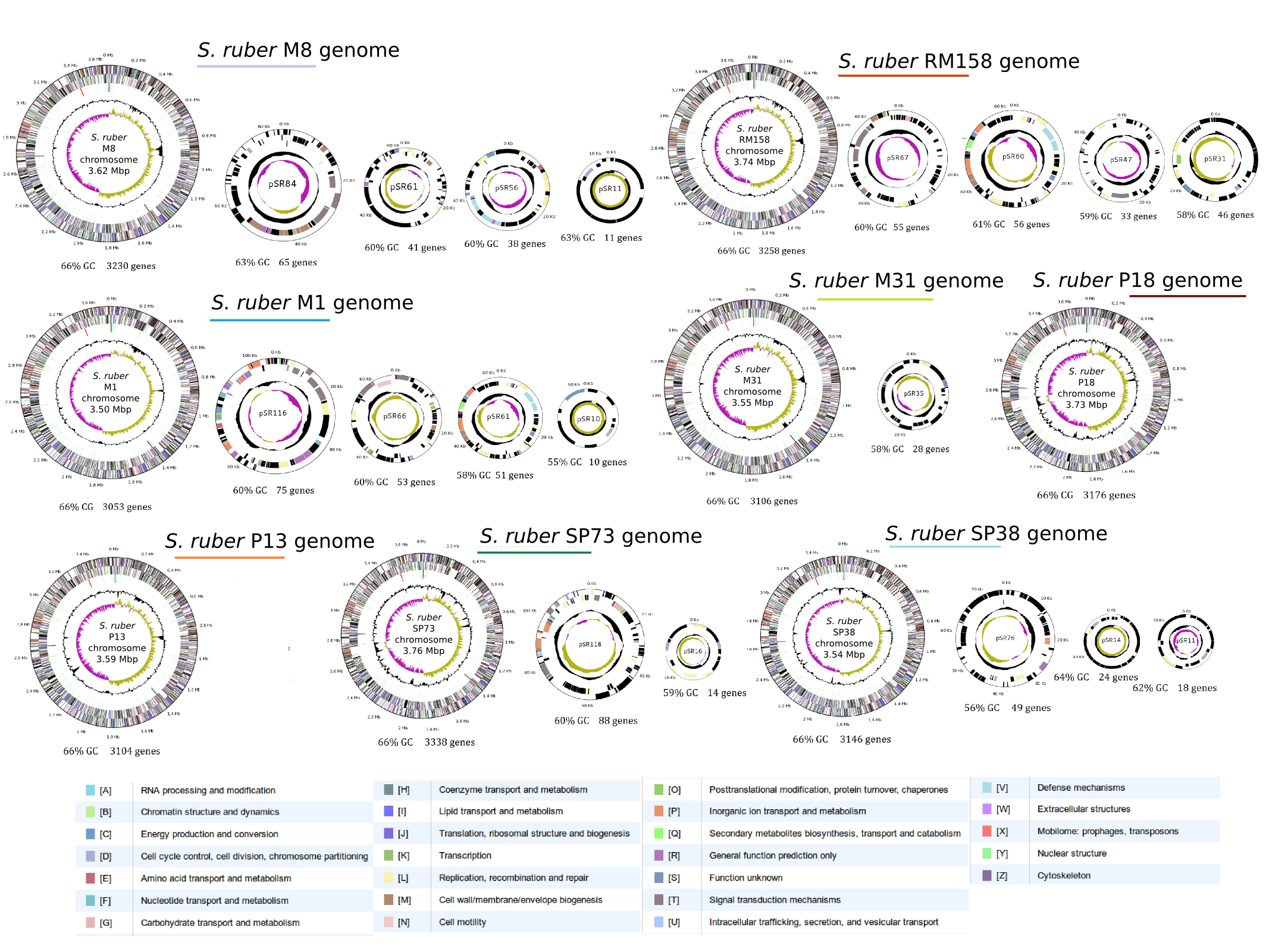

Supplement: FIGURE S1 — Representation of 24 replicons (8 chromosomes and 18 plasmids) present in the eight strains in this study. The main traits (nomenclature, genome size, gene and GC content) are detailed for each genome. From inner to outside ring: Ring 1: represents GC skew index (GCSI) defined as (G-C/C+C) (green and purple); Ring 2: GC content (%) (colored in black). Genes appears represented in external rings and colored based on clusters of orthologous groups (COGs) functional classification. Colors corresponds to those associated to each strain in Figure 1. [file Image_1.TIFF]

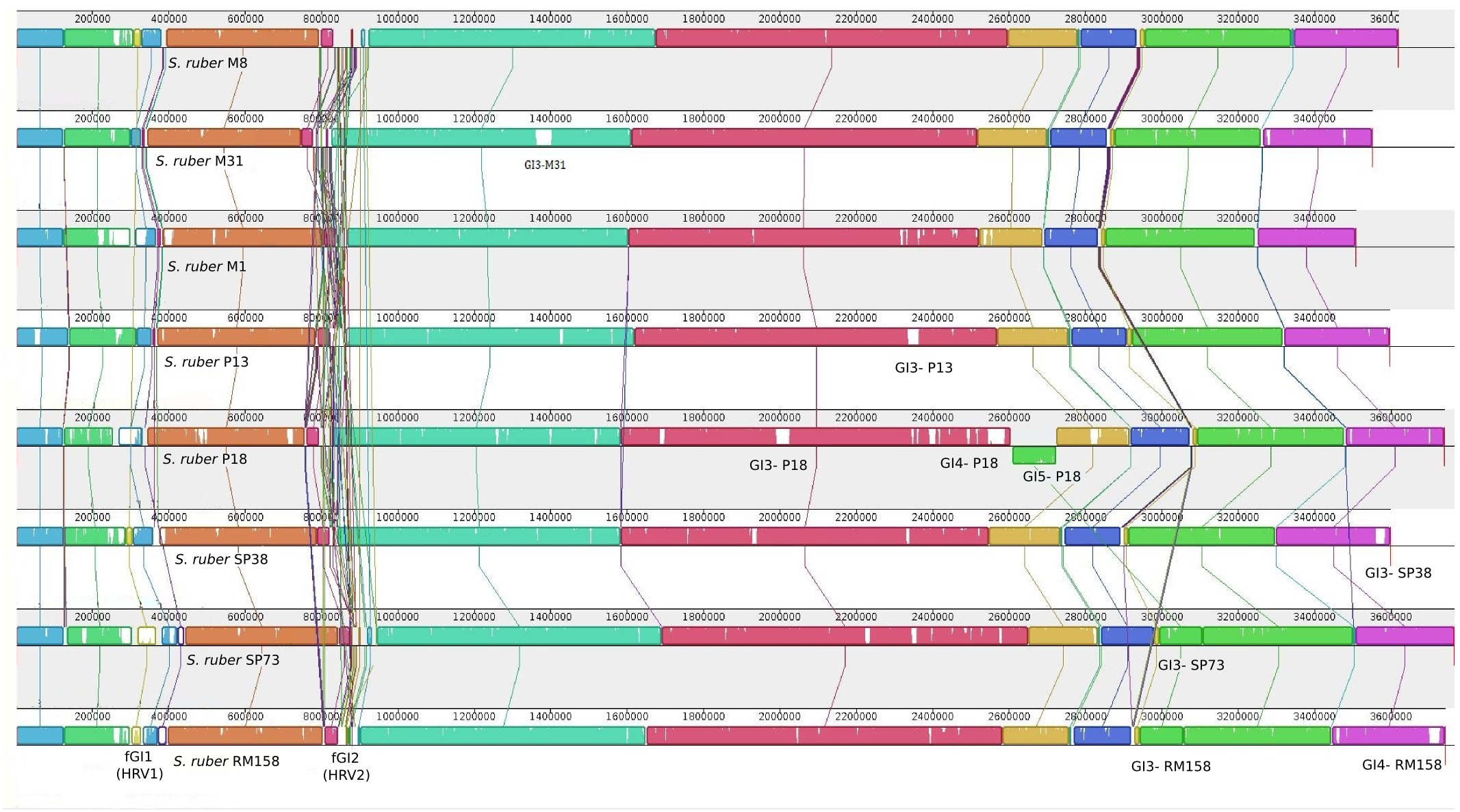

Supplement: FIGURE S2 — Chromosome alignment representation for the eight S. ruber strains considered in this study. Extensive syntenic core genome regions appears represented as collinear blocks interrupted by accessory elements; genomic islands and indels. Colored fraction inside each block is proportional to the sequence identity level with respect to M8 strain. [file Image_2.TIFF]

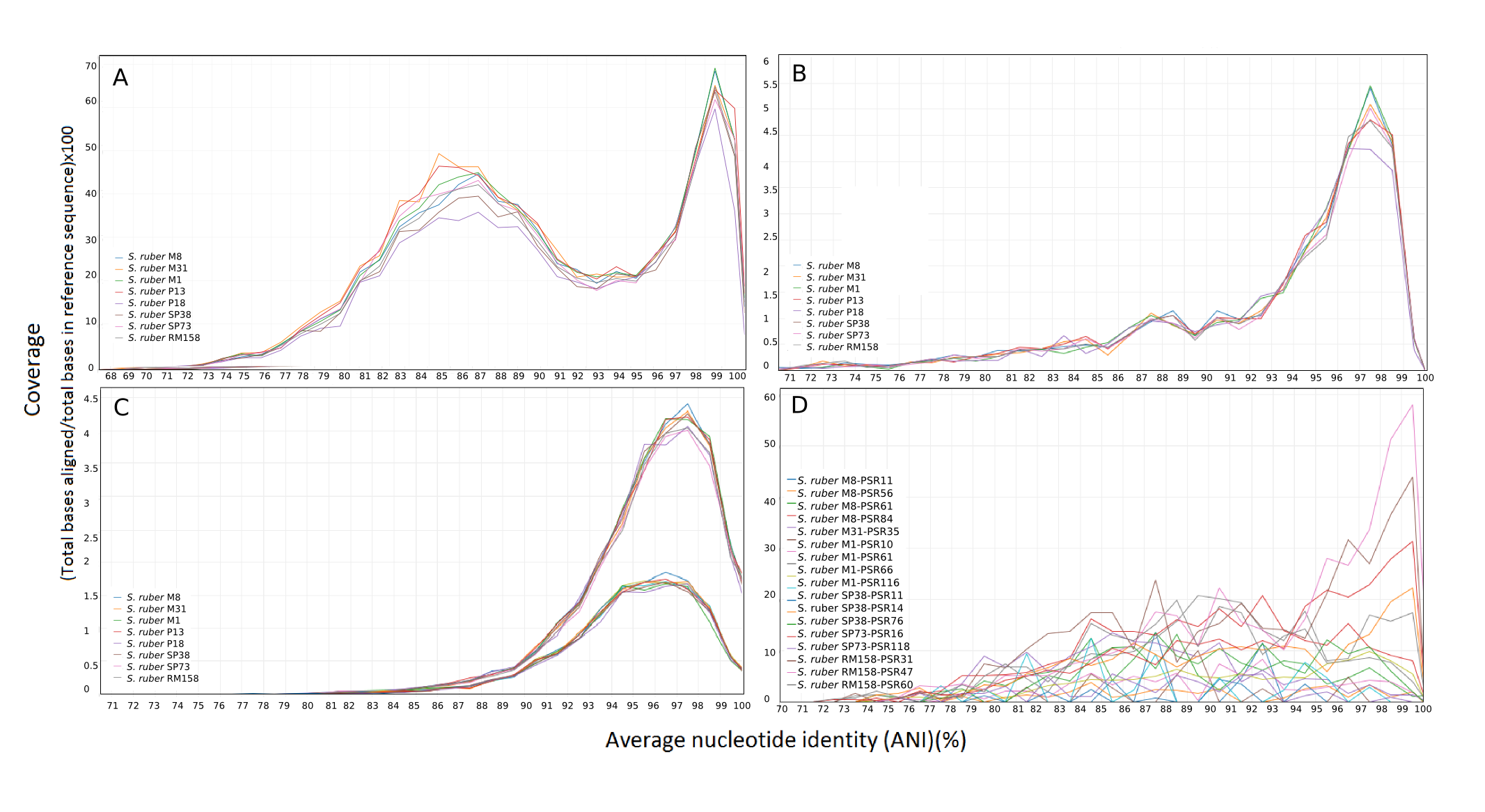

Supplement: FIGURE S3 — Metagenomic recruitment and discrete populations. Coverage plot representing fragment recruitment of the (A) Santa Pola CR30 crystallizer (Spain) (Fernandez et al., 2013); (B) Lake Tyrrell metagenome (Australia) and (C) two different San Diego High Salt ponds metagenomes (United States) against S. ruber main chromosomes and (D) Santa Pola CR30 crystallizer (Spain) (Ghai et al., 2011) against plasmids sequences. For each graph, the coverage (y-axis) is normalized to the length of the reference and represents the relative in situ abundance of the corresponding population. [file Image_3.TIFF]

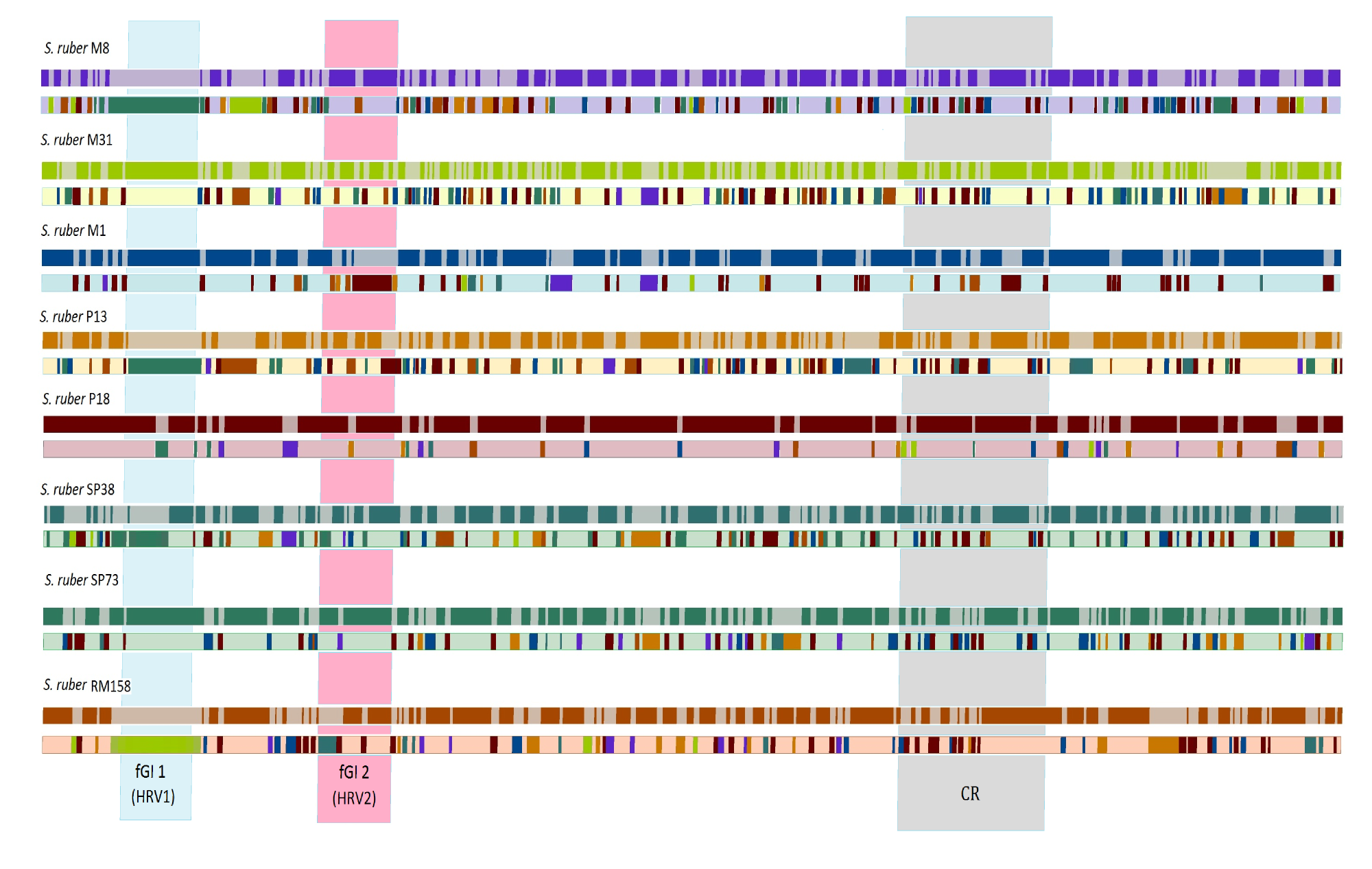

Supplement: FIGURE S4 — Homologous recombination map. Genomic map representing the inferred recombination events among the considered strains. Each strain appears associated to a color code (top bar) consistent with Figure 1 and Supplementary Figure S1. The bottom bars represent the recombination events, which are colored according to the predicted donor strain. FGI1, fGI2 and the conservated region (CR) described in previous studies (Peña et al., 2010) are highlighted in the figure. [file Image_4.TIFF]

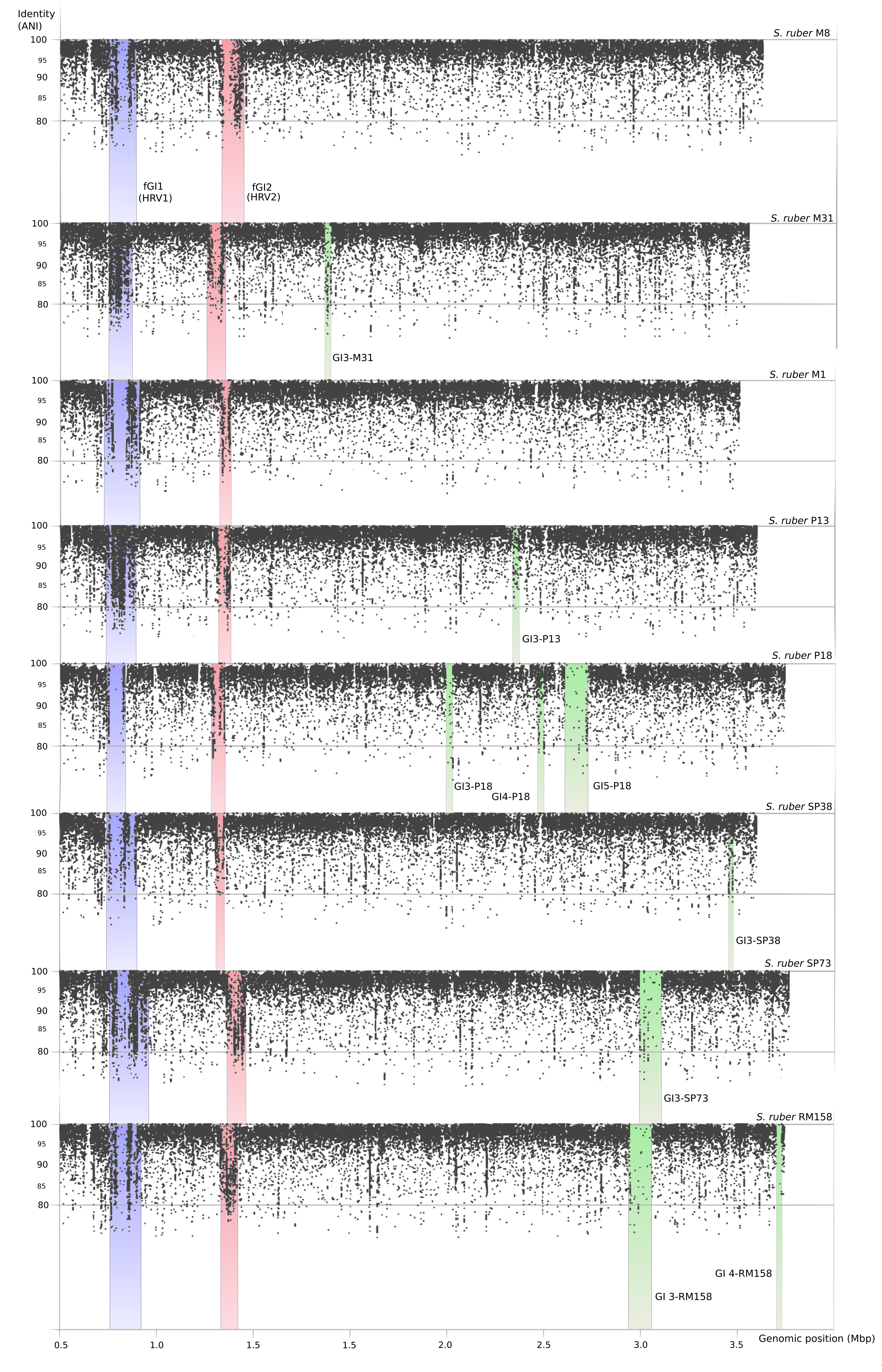

Supplement: FIGURE S5 — Metagenomic recruitment and GI identification. Alignment of the eight S. ruber chromosome sequences with metagenomic sequences from Santa Pola saltern pond, CR30 crystallizer (Spain) (Ghai et al., 2011). Values on the y-axis indicate nucleotide percent identity, usually over 95%. Areas with unusually low representation in the metagenome corresponded to under-recruiting genomic islands: fGIs1 and fGIs2 (indicated in blue and green, respectively) and HGT-GIs in green. [file Image_5.TIFF]

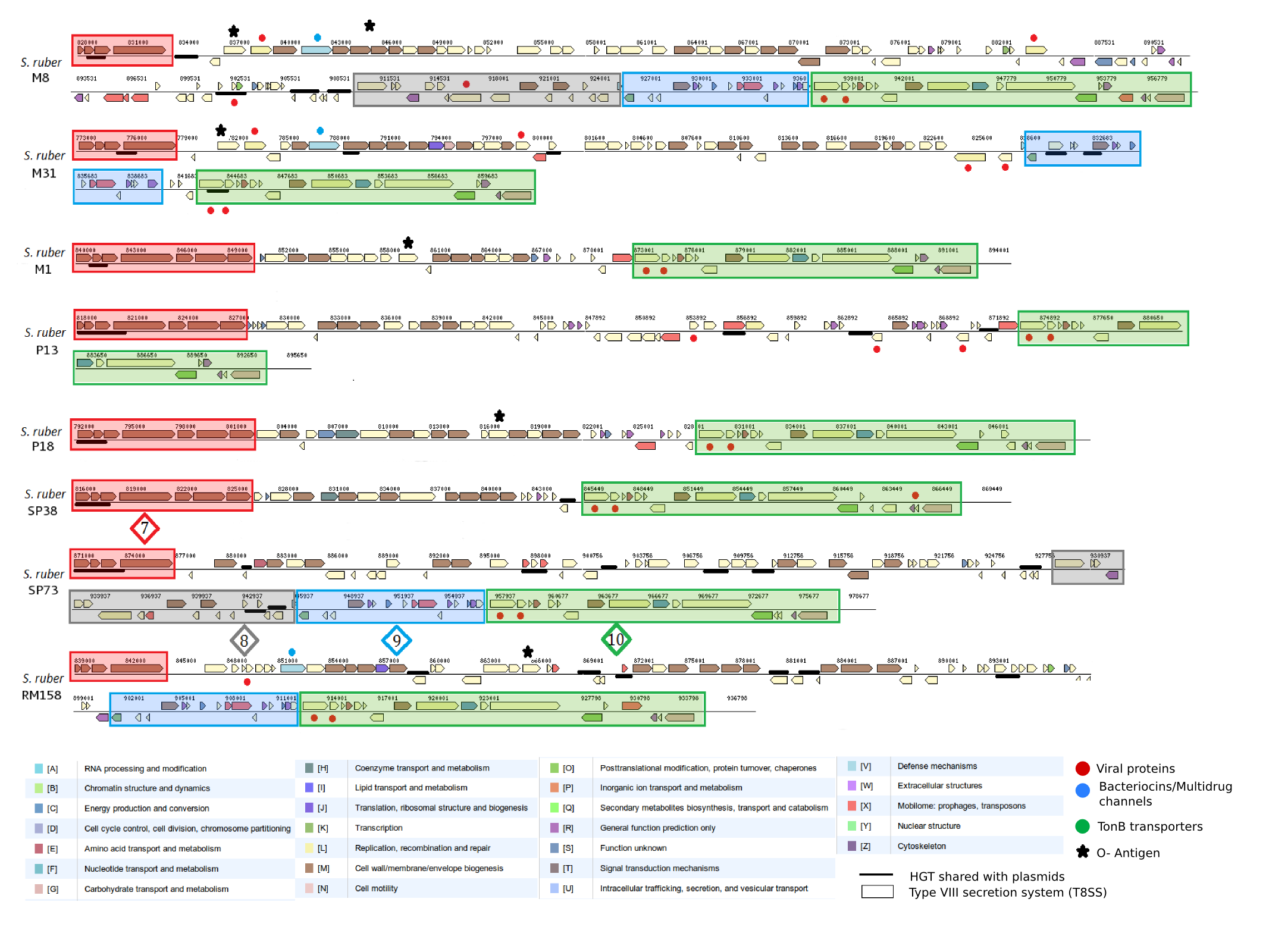

Supplement: FIGURE S6 — Overall comparison of the genomic architecture, gene content and clusters distribution, of fGI2 (HRV2) among the eight analyzed strains. Regions shared with plasmids are underlined. Six different syntenic clusters or cassettes were identified and delimited in numbered colored boxes. Gene colors and symbols were consistent with Figure 4. [file Image_6.TIFF]

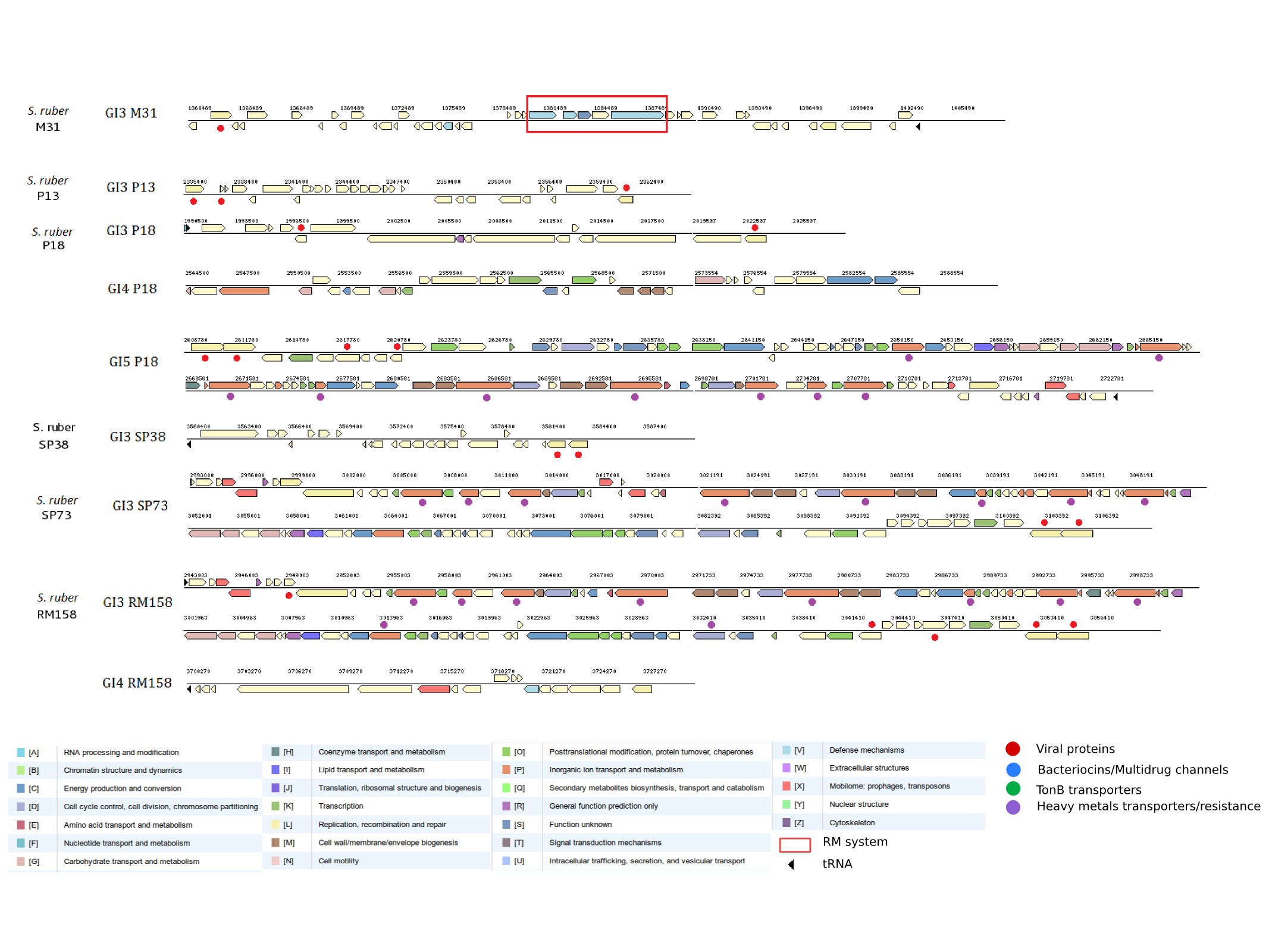

Supplement: FIGURE S7 — Gene content and functional traits of HGT-GI islands among the eight analyzed strains. RM systems are delimited with red boxes and tRNA genes are labeled with triangles. Gene colors and symbols were consistent with Figure 4. [file Image_7.TIFF]

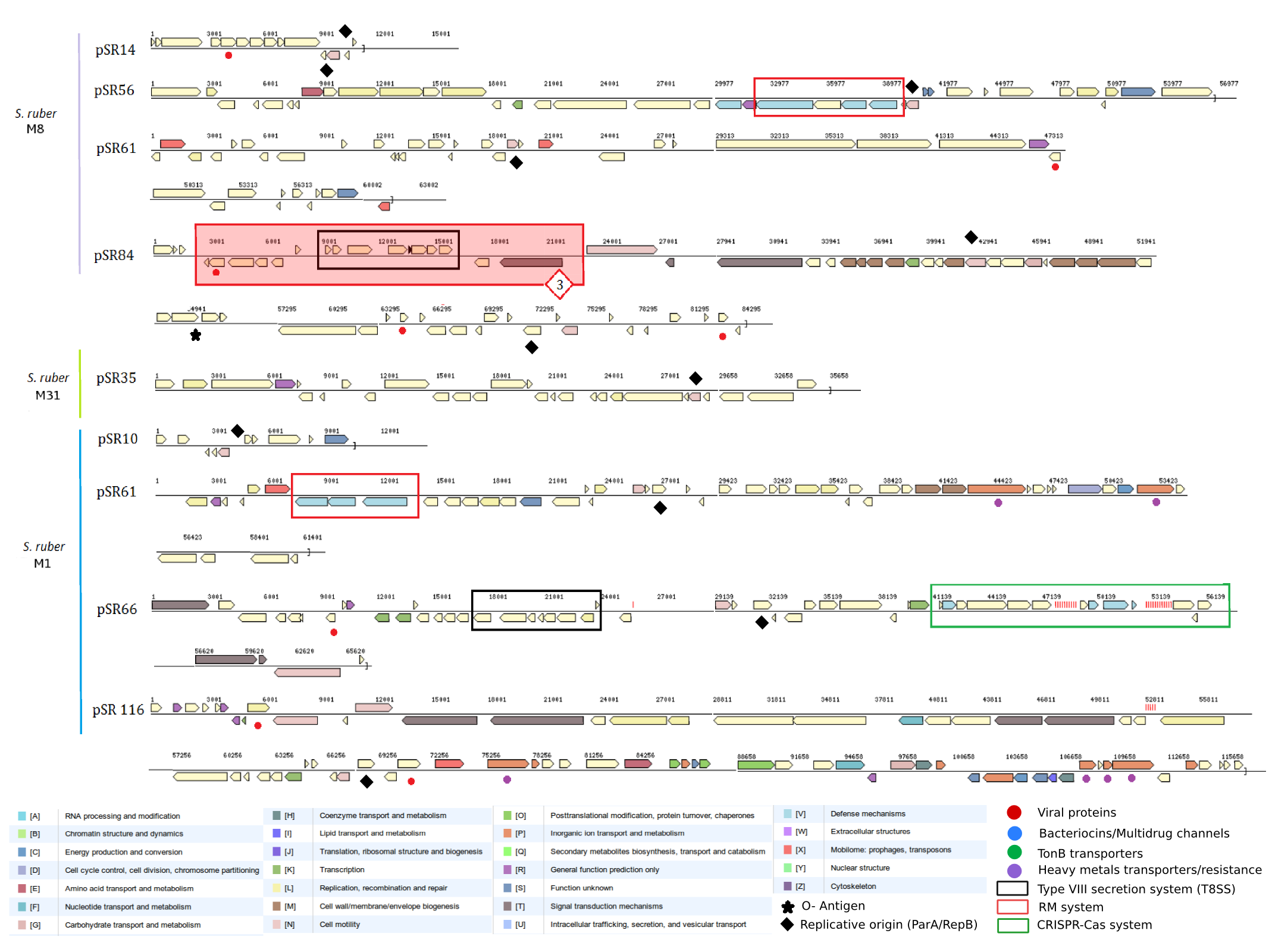

Supplement: FIGURE S8 — Gene content and functional traits of plasmids detected amongst three of the eight strains analyzed in this study (S. ruber M8, S. ruber M31, S. ruber M1). RM systems, type 8 secretion systems (T8SS) and CRISPR-Cas systems are delimited in boxes and genes are labeled with a triangle and appear flanking HGT-GI islands. Three different events were identified and delimited in numbered color filled boxes representing three cases of extensive homologous regions involving plasmids of four different strains: M8 (pSR84), SP73 (pSR118), SP38 (pSR76), and RM158 (pSR60, pSR67). Gene colors and symbols were consistent with Figure 4. [file Image_8.TIFF]

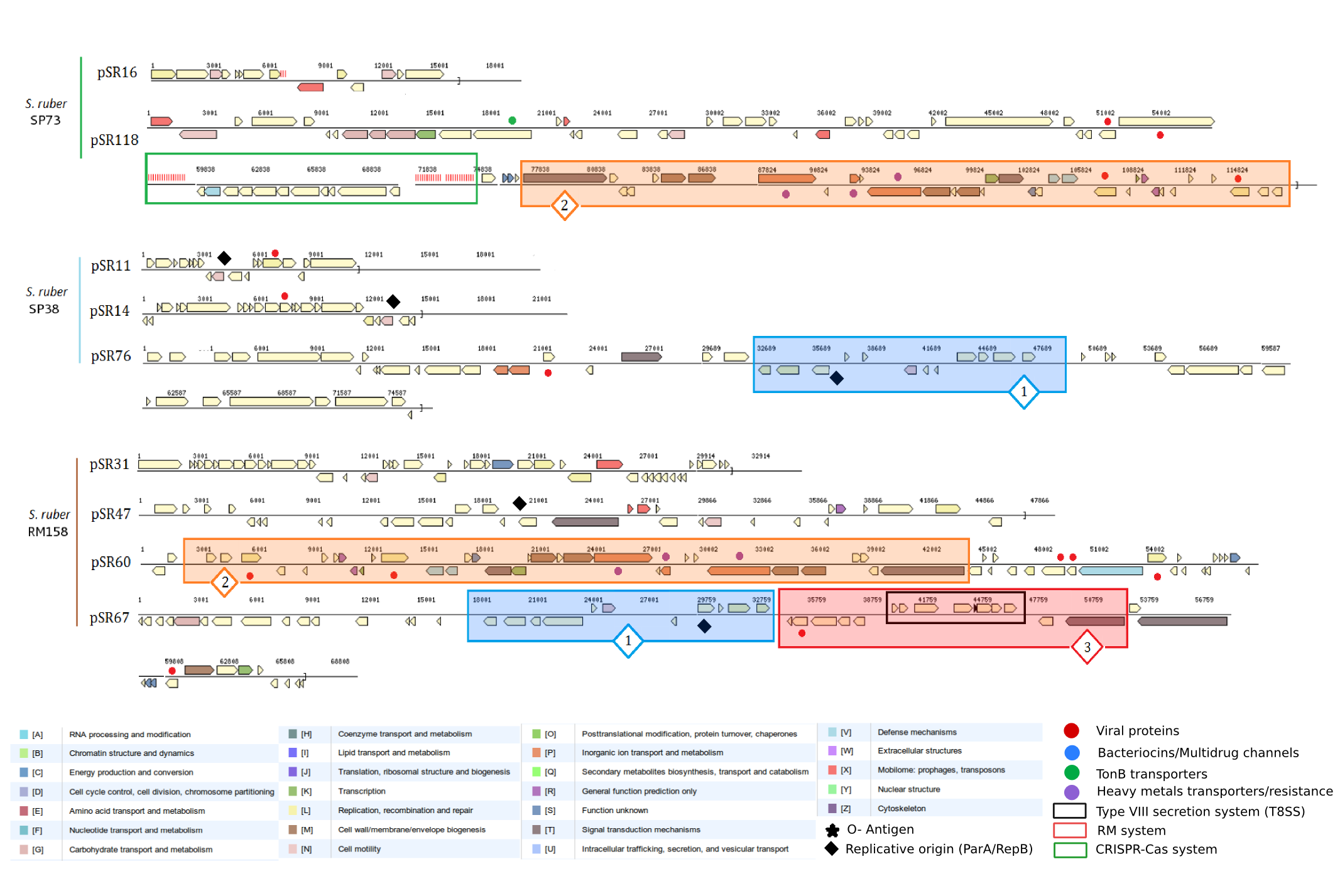

Supplement: FIGURE S9 — Gene content and functional traits of plasmids detected amongst three of the eight strains analyzed in this study (S. ruber SP73, S. ruber M38, and S. ruber RM158). RM systems, type 8 secretion systems (T8SS) and CRISPR-Cas systems are delimited in boxes and genes are labeled with a triangle and appear flanking HGT-GI islands. Three different events were identified and delimited in numbered color filled boxes representing three cases of extensive homologous regions involving plasmids of four different strains: M8 (pSR84), SP73 (pSR118), SP38 (pSR76), and RM158 (pSR60, pSR67). Gene colors and symbols were consistent with Figure 4. [file Image_9.TIFF]

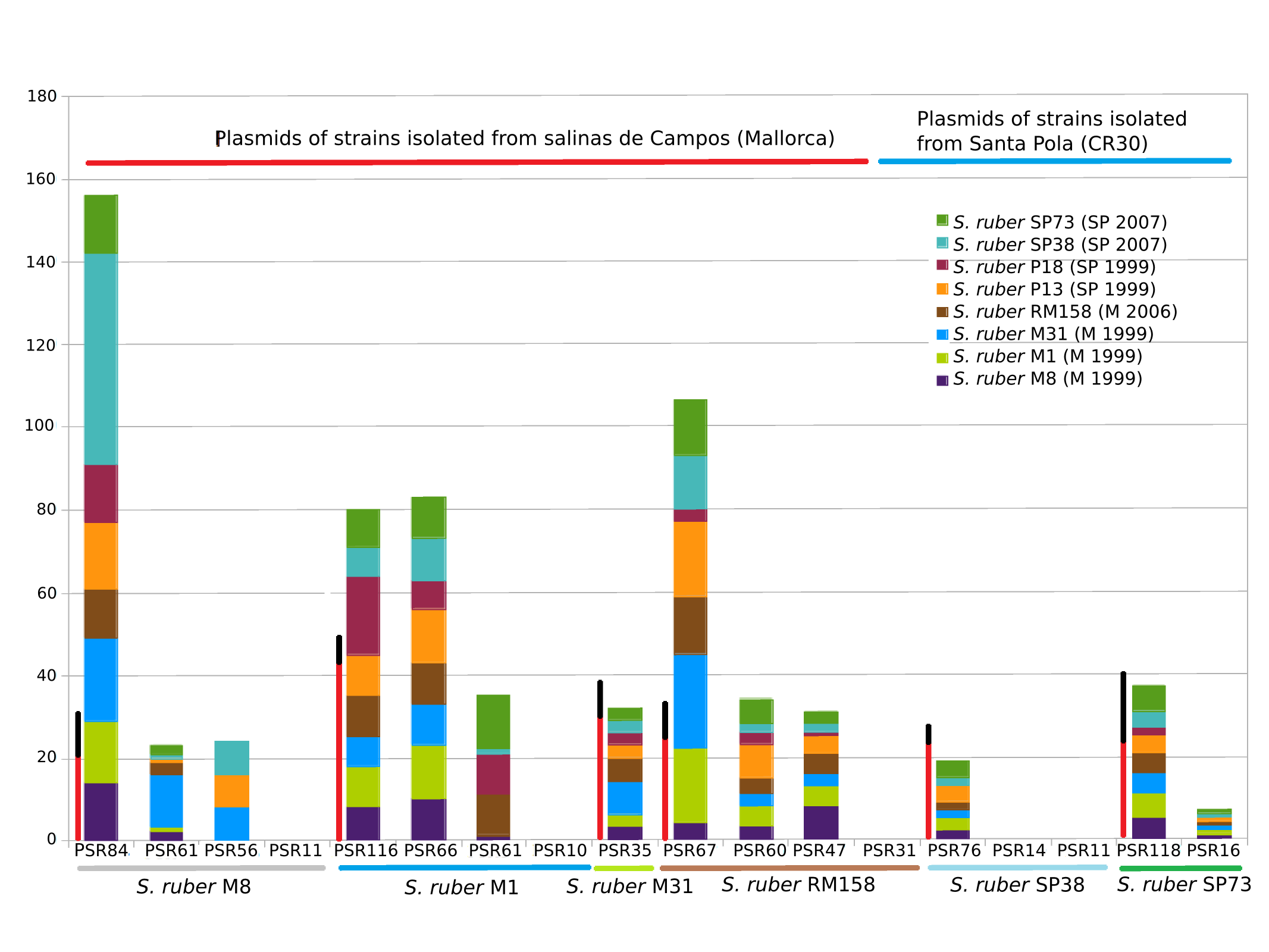

Supplement: FIGURE S10 — The main bar represents the cumulative percentage (y-axis) of 18 plasmid sequences, included in the genome of six S. ruber strains (x-axis), that presented homology with chromosome sequences among the eight different S. ruber strains (colored in the legend). Among these sequences, an additional unique secondary bar per strain represents the percentage of total sequences that presented homology with fGI1 (in red) and fGI2 (in black). For P13 and P18 strains (values not represented), the last distributions were 37.11 and 4.6% for fGI1 and 42.26 and 14.81% for fGI2, respectively. [file Image_10.TIFF]

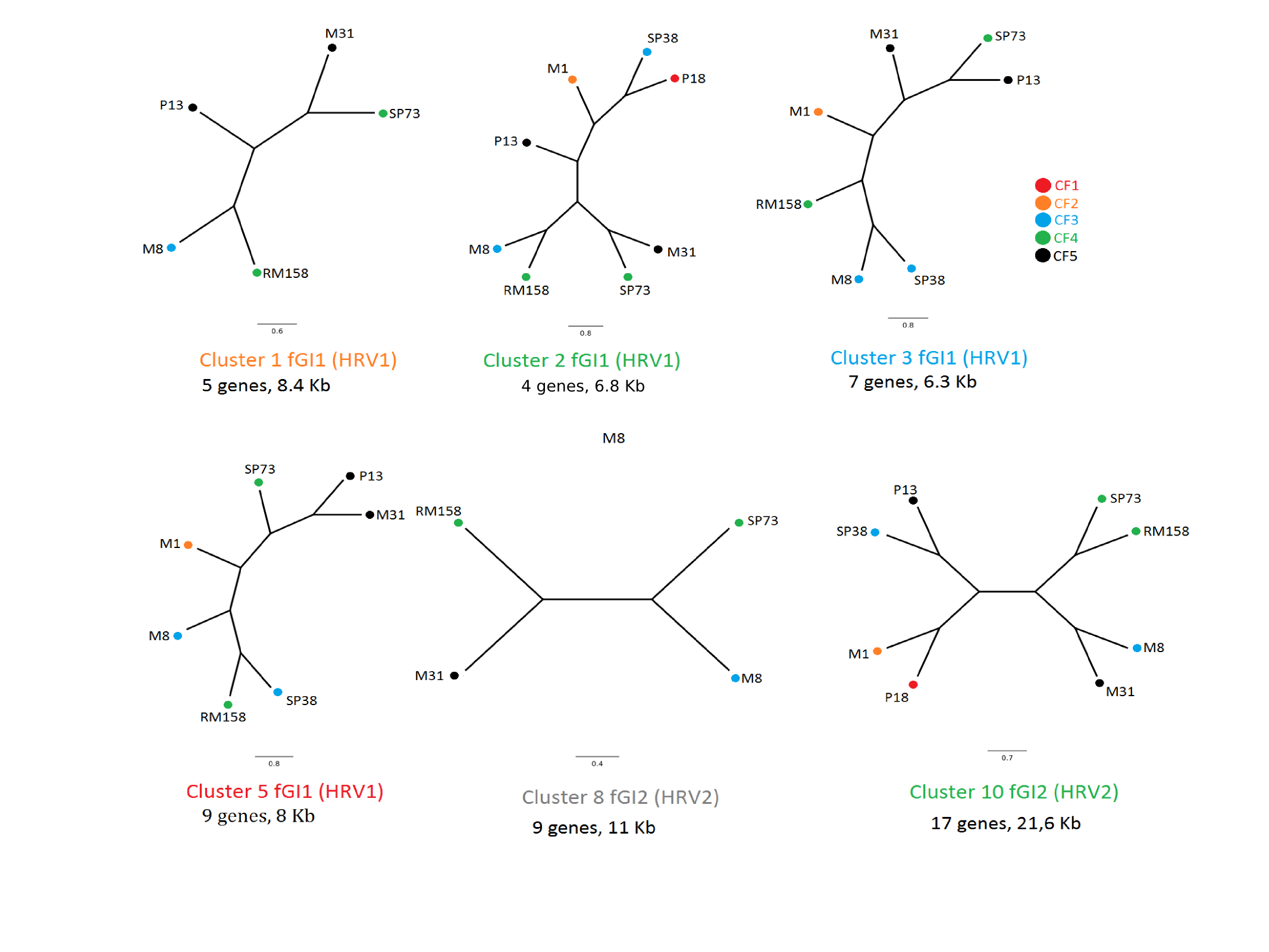

Supplement: FIGURE S11 — ML trees of 6 of the 10 syntenic clusters or cassettes shared by at least four strains and described in fGI1 and fGI2 (Figure 4 and Supplementary Figure S3). Strains are indicated in all the branches and clonal frames (CFs) correspondence indicated by colored dots consistent with Figure 4. The line segment at the bottom part of each tree shows the branch length scale (substitutions/site units). [file Image_11.TIFF]

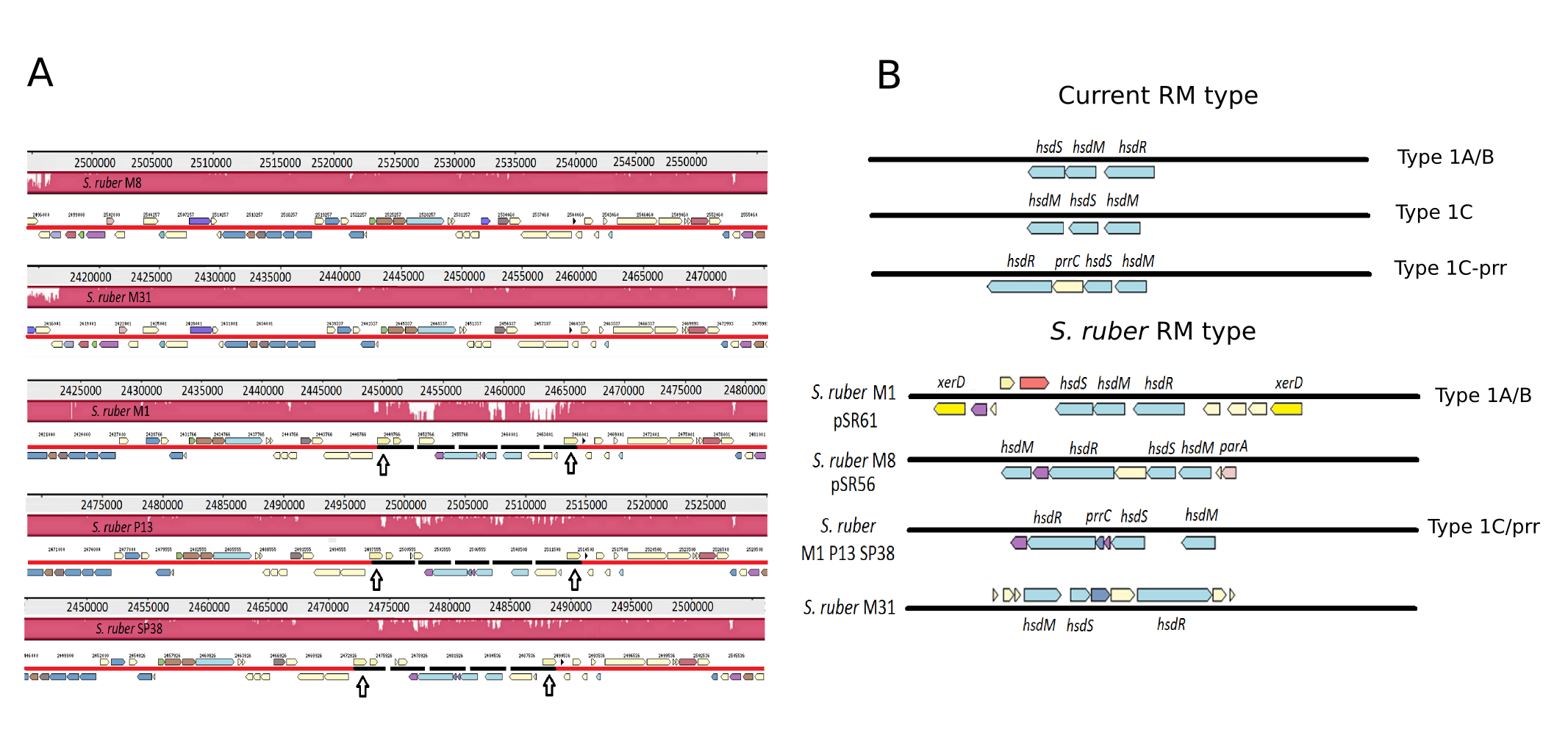

Supplement: FIGURE S12 — RM systems diversity and microevolutive mechanisms. (A) Genome alignment showing the syntenic genomic environment (underlined in red) interrupted in case of S. ruber M1, SP38 and P13 strains by an indel (underlined in back) that contained a type 1C-prr RM system, flanked by tirosin recombinases XerD, likely involved in the chromosome integration. (B) Most common RM systems present in bacteria (upper part) and those present amongst S. ruber strains (bottom). [file Image_12.TIFF]

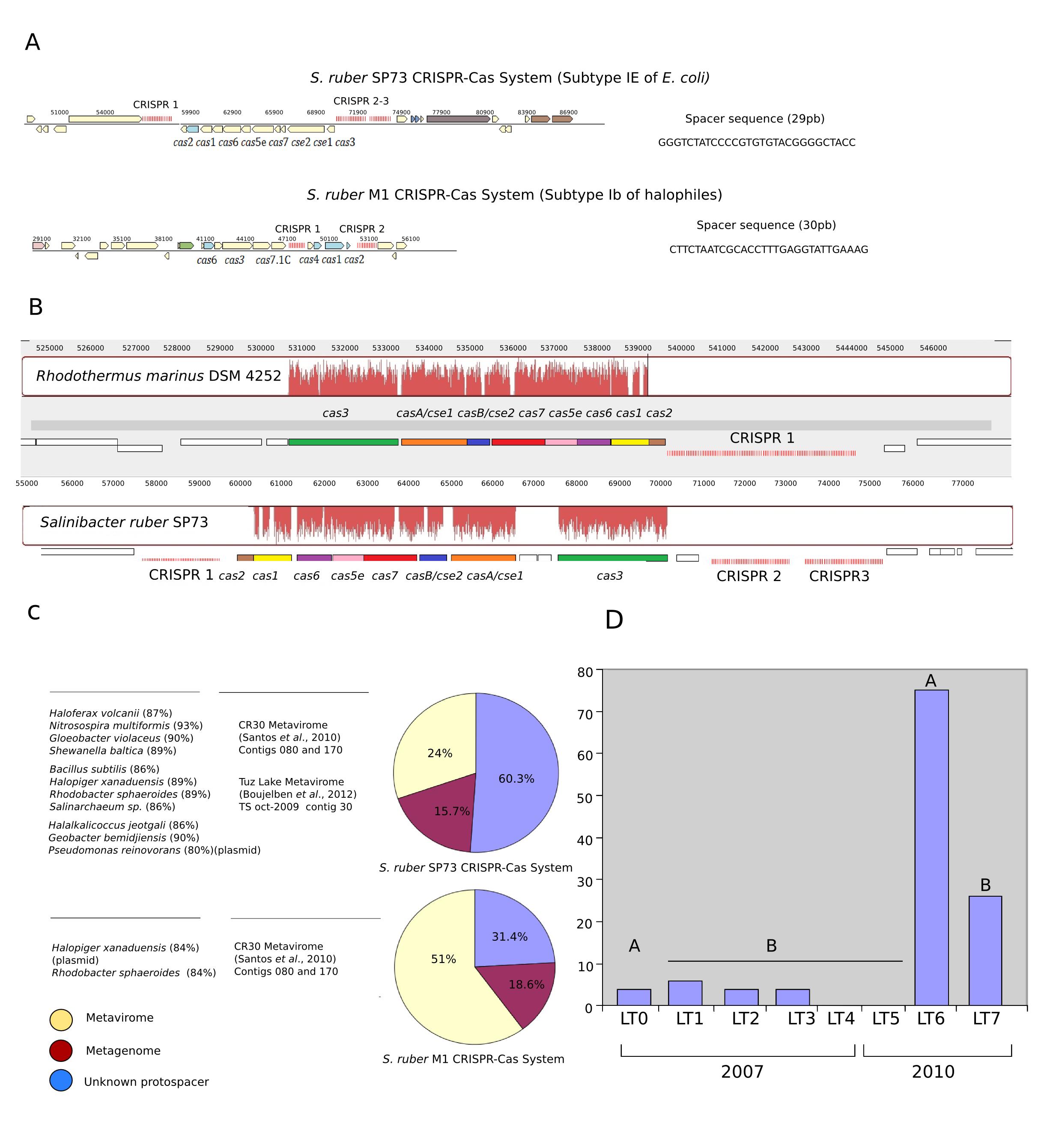

Supplement: FIGURE S13 — CRIPR-Cas systems microdiversity. (A) CRISPR- Cas systems found in S. ruber SP73 and S. ruber M1 plasmids, indicating the type and cas genes content and spacer sequence. CRISPR arrays were represented with dotted red lines. (B) Comparison and syntenic similarities between CRISPR-Cas system found in S. ruber SP73 strain and its best hit in Rhodothermus marinus DSM 4252 strain. (C) Distribution of the main protospacer hits amongst environmental metagenomes (yellow), metaviromes (red), and unknown (blue). (D) Number of hits found mean protospacer recruitment versus lake Tyrrell Site A and B metaviromes from 2007 and 2010 years. [file Image_13.TIFF]
